# Resistance-containing exercise versus non-exercise control for inflammatory markers and T-cell counts in adults living with HIV: a systematic review and meta-analysis

**DOI:** 10.3389/fimmu.2026.1949799

**Published:** 2026-10-07

**Authors:** Jiayi Zhang, Yafei Gao, Zhentao Zhang, Zikun Lyu, Jingshi Ma

**Affiliations:** Department of Physical Education, Kunsan National University, Gunsan-si, Republic of Korea

**Keywords:** CD4-positive T-lymphocytes, combined exercise, cytokines, HIV, inflammation, meta-analysis, resistance training, systematic review

## Abstract

**Background:**

Adults living with HIV may have persistent immune activation and low-grade inflammation despite effective antiretroviral therapy. We assessed whether resistance training or resistance-containing combined exercise alters inflammatory biomarkers or T-cell counts versus non-exercise control.

**Methods:**

Six databases were searched to 1 June 2026 without language restrictions. Controlled intervention studies lasting at least four weeks were eligible. Screening and extraction were performed independently in duplicate. Inflammatory outcomes were expressed as Hedges’ g; final-value SMDs were primary and change-score SMDs were analyzed separately, with one-trial strata reported individually. CD4+ T-cell count was pooled as a mean difference. Random-effects models used restricted maximum likelihood and Hartung-Knapp-Sidik-Jonkman intervals. RoB 2 or ROBINS-I and GRADE were applied.

**Results:**

Fifteen trials (18 reports) were included; 13 contributed quantitative data. All primary final-value confidence intervals included no effect: interleukin-6, −0.19 (95% CI −1.14 to 0.76; k = 4); tumor necrosis factor alpha, 0.07 (−1.44 to 1.57; k = 3); C-reactive protein, −0.02 (−1.46 to 1.41; k = 3); interleukin-1 beta, −0.47 (−10.09 to 9.15; k = 2); and interleukin-10, 0.04 (−11.37 to 11.46; k = 2). Interleukin-8 was reported by one final-value trial, −0.63 (−1.39 to 0.12), and was not pooled. Separate change-score analyses were also inconclusive except for one unpooled C-reactive protein trial. The pooled estimate for CD4+ T-cell count favored exercise by 124.2 cells/mm^3^ (54.6 to 193.7; k = 7), but the prediction interval crossed no effect. Certainty was very low.

**Conclusion:**

The effects of resistance-containing exercise on inflammatory biomarkers remain undetermined. The CD4+ estimate suggests a possible increase, but confidence is very limited because the evidence is sparse, heterogeneous, at risk of bias, and its prediction interval includes no effect. Larger trials with well-defined comparators and complete contrast-form reporting are needed.

**Systematic review registration:**

https://www.crd.york.ac.uk/PROSPERO/view/CRD420261425700, identifier CRD420261425700.

## 1 Introduction

Antiretroviral therapy has transformed HIV infection from a progressive, usually fatal illness into a chronic condition compatible with a near-normal lifespan (1, 2). That success has revealed a second problem. Even among adults with durable viral suppression and restored CD4+ T-cell counts, markers of immune activation and low-grade systemic inflammation frequently remain above the levels seen in people without HIV (1, 3). This residual inflammatory state is not a laboratory curiosity. Elevated interleukin-6, C-reactive protein and D-dimer concentrations predict all-cause mortality and non-AIDS events in treated cohorts (4, 5), soluble inflammatory markers outperform T-cell activation markers in forecasting non-AIDS morbidity during suppressive therapy (6), and the same biology is implicated in the excess burden of cardiovascular, metabolic and skeletal disease observed in this population (7, 8). Reducing residual inflammation has therefore become an explicit therapeutic target alongside virological control.

The mechanisms that sustain this state are several and partly independent of viral replication: microbial translocation across a damaged gut mucosa, persistent low-level antigen stimulation, thymic dysfunction, co-infections and the cumulative toxicity of long-term therapy (3). Because no single pharmacological agent addresses this cluster, attention has turned to interventions that act broadly on the inflammatory milieu. Exercise is the most prominent of these. Regular physical activity lowers circulating inflammatory markers in a range of chronic conditions, and the proposed mechanisms — reduction in visceral adipose tissue and its adipokine output, and the acute anti-inflammatory environment created by each bout of contraction, mediated in part by muscle-derived signaling molecules — are plausible in HIV (9–11).

Resistance training has a particular claim in this setting. Loss of skeletal muscle mass and function is common in long-term HIV infection, and skeletal muscle is itself an endocrine organ whose secretory output changes with loading (11). Resistance exercise is thus advocated both to counter the muscle-specific consequences of infection and therapy and, more speculatively, to modify the inflammatory state directly. Trials in adults living with HIV have accordingly examined resistance training alone and combined programs containing a resistance component, measuring cytokine panels and immune cell counts alongside functional endpoints.

The evidence these trials have produced is not easy to read. Existing systematic reviews of exercise in HIV have concentrated on cardiorespiratory fitness, strength, body composition and psychological outcomes, treating immunological endpoints as secondary (12–15). Where inflammatory outcomes have been synthesized, reviews have tended to combine aerobic and resistance modalities, or to pool across biomarkers whose biology and measurement differ (16, 17). Individual trials are small, and their reported effects for the same biomarker range from substantial reduction to substantial increase; one trial found that adding resistance training to inpatient care improved skeletal muscle health without altering cytokine concentrations at all (18). Physical activity levels in this population are low (19), which makes the question practically as well as biologically consequential.

Three features of this literature warrant a synthesis designed specifically around them. First, the outcomes are chemically and biologically distinct constructs, so pooling should be organized by construct rather than by an aggregate notion of “inflammation”. Second, trials differ in whether they report post-intervention values or change from baseline. Standardized effects derived from these forms use different standardizing standard deviations and are not directly comparable, so they require separate synthesis. Third, comparators vary from genuine no-exercise control to recreationally active control and to placebo, medication, or care backgrounds applied equally in both arms; this variation may contribute to between-trial differences but does not necessarily make the exercise contrast indirect.

We therefore conducted a systematic review and meta-analysis to determine the effects of structured resistance training or resistance-containing combined exercise, compared with a non-exercise comparator, on inflammatory biomarkers and T-cell counts in adults living with HIV, synthesizing each construct separately. For the inflammatory constructs, final-value standardized mean differences were designated as the primary evidence and change-score standardized mean differences as supporting evidence; variation by study characteristics was examined only in *post hoc* exploratory analyses.

## 2 Methods

### 2.1 Reporting guidance

This review is reported in accordance with the PRISMA 2020 statement and its explanation and elaboration document (20, 21), and the search is reported in accordance with the PRISMA-S extension (22). Methods followed the Cochrane handbook for systematic reviews of interventions (23, 24). The review protocol was registered in PROSPERO (registration number CRD420261425700). The separation of final-value and change-score standardized mean differences, the designation of final values as primary, and all subgroup and robustness analyses were not specified in the registered protocol and are therefore reported as *post hoc* exploratory analyses.

### 2.2 Eligibility criteria

Studies were eligible according to the following criteria.

Population. Adults aged 18 years or older living with HIV, regardless of antiretroviral regimen or treatment status.

Intervention. Structured resistance training, or combined/concurrent exercise containing an identifiable resistance component, lasting at least four weeks.

Comparator. A non-exercise comparator: usual care, waiting list, recreational or health-education control, or a shared medication background without exercise.

Outcomes. Inflammatory biomarkers and T-cell measures defined the review question; the quantitative outcome set comprised interleukin-6, tumor necrosis factor alpha, C-reactive protein, interleukin-1 beta, interleukin-8, interleukin-10, and CD4+ T-cell count. The secondary outcome set, detailed in **Supplementary Table S7**, included additional inflammatory and T-cell measures, HIV-1 viral load, growth factors, adipokines, neurotrophic factors and hormones; safety and adherence were also assessed narratively. The secondary outcomes were listed at the eligibility stage so that each report was screened once against the complete outcome set, and they were prespecified for narrative rather than quantitative synthesis; they are reported in Section 3.8, with the reason for narrative synthesis recorded for each in the **Supplementary Material**.

Study designs. Randomized controlled trials, quasi-randomized controlled trials, and non-randomized controlled intervention studies with a parallel non-exercise comparator.

Studies were excluded when the comparator was an active exercise arm with no non-exercise arm; when a multicomponent intervention could not be isolated to its exercise element; when only acute (single-bout) exercise was studied; when the design was uncontrolled; and when outcome data contained internal errors that could not be recovered.

The unit of synthesis was the trial. Where several reports described the same trial, they were merged under a single trial identifier and counted once.

### 2.3 Information sources and search strategy

Six databases were searched from inception to 1 June 2026: PubMed, Web of Science Core Collection, Embase, Cochrane Library, Scopus and SPORTDiscus. No language restriction was applied. The search combined four concept blocks — HIV, resistance or combined exercise, inflammatory and immunological outcomes, and controlled trial design — using database-appropriate controlled vocabulary and free-text terms. Reference lists of included reports and of prior reviews were inspected for additional records. The database-specific search strategies, together with the recorded search date and number of records retrieved from each database, are reported in **Supplementary Table S1**.

Trial registries were not searched as a separate information source, authors of included reports were not contacted, and the search strategy was not peer reviewed by a second information specialist. Registry records were covered in part through the Cochrane Library (CENTRAL), which incorporates records from ClinicalTrials.gov and the World Health Organization International Clinical Trials Registry Platform; however, this indirect coverage is not equivalent to direct registry searching, and unpublished or ongoing studies may therefore have been missed. Because fewer than 10 trials contributed to every pooled construct, funnel-plot methods were not used and small-study effects cannot be excluded. This limitation is considered further in Section 4.5. This review was not an update of an earlier review.

### 2.4 Selection and data collection

Records were deduplicated and then screened on title and abstract against the eligibility criteria by two reviewers (JZ and YG) working independently; a record retained by either reviewer proceeded to full text. Full-text reports were assessed for eligibility by the same two reviewers, again independently and in duplicate. Data were extracted independently and in duplicate by the same two reviewers into a piloted extraction form, and the two records were compared item by item. Disagreements at the screening, eligibility and extraction stages were resolved by discussion between the two reviewers, and a third reviewer (JM) adjudicated any that remained; the third reviewer’s decision was final. Every extracted value was additionally checked against the source report by the third reviewer in a separate source-verification step, in which the numeric values, group denominators, units, contrast form and outcome labels were compared with the corresponding table, figure or text in the original publication. Discrepancies identified at verification were resolved by returning to the source report.

Extracted items comprised study identifiers and design; country; participant characteristics including age, sex distribution and baseline clinical status; the content, duration, frequency, session length, intensity prescription, progression rule, supervision and delivery mode of the intervention; the nature of the comparator; adherence; the timing of outcome assessment; and, for every outcome, the reported group means, dispersion measures, denominators, units and contrast form.

### 2.5 Outcome constructs

The outcome-level data used to compute every effect estimate, including the group means, dispersion measures, denominators, units, contrast form and the derivation of any reconstructed dispersion, are reported in full in **Supplementary Table S2**.

Outcomes were standardized to clinical-biological constructs before synthesis. Values reported under different assay-specific labels or in convertible units were mapped onto a single construct where they measured the same analyte, and distinct analytes were never merged. Original outcome labels were retained for traceability but were not used as analysis units. For a primary inflammatory construct, two or more independent trials within the same contrast form were required for meta-analysis; a stratum containing one trial was reported as an individual study result. The prespecified secondary outcomes remained narrative regardless of study count, as specified in Section 2.2. Analysis roles are defined consistently throughout this review: Primary denotes final-value estimates for the six quantitative inflammatory constructs and the combined-form CD4+ mean-difference analysis; Supporting denotes the separate inflammatory change-score estimates; Sensitivity denotes a *post hoc* robustness check of a Primary or Supporting reference analysis; and Narrative denotes descriptive reporting without cross-trial pooling. A one-trial Primary or Supporting stratum retains that role and is reported individually, whereas Narrative outcomes may have individual contrasts tabulated without being meta-analyzed.

### 2.6 Effect measures

Each construct was analyzed on a single effect metric, and standardized and unstandardized estimates were never combined within an analysis. CD4+ T-cell count was expressed as a mean difference in cells/mm^3^. Every inflammatory biomarker construct was expressed as a standardized mean difference using Hedges’ g with the small-sample bias correction (25). Final-value and change-score standardized effects were not combined. Final-value standardized mean differences were designated as primary because they provided a common standardization basis across the construct set without requiring an unavailable within-person pre-post correlation; change-score standardized mean differences were synthesized separately as supporting evidence. For CD4+ T-cell count, final-value and change-score effects were combined as unstandardized mean differences because both estimate the absolute between-group difference in the same unit. All contrasts were oriented as exercise minus comparator. Directions were harmonized before analysis so that, for the pro-inflammatory constructs, a negative value indicates lower concentrations in the exercise group; for CD4+ T-cell count a positive value indicates a higher count in the exercise group. Interleukin-10 was treated as directionally neutral, because a higher concentration is interpretable as anti-inflammatory or pro-inflammatory depending on context.

Where a trial reported dispersion in a form other than the standard deviation, the standard deviation was reconstructed using the standard relationships set out in the Cochrane handbook (23), and the derivation was recorded for each affected estimate. Trials contributing more than one eligible stratum or arm were handled so that no participant group entered a single pooled estimate more than once. One trial, Soares et al. (51), had two eligible exercise arms sharing a single comparator group. Its two exercise arms were combined into one group using the Cochrane handbook formulae for combining means and standard deviations across groups (23) before the contrast was formed, so the comparator entered the analysis once and at its full size; dividing the comparator was therefore not required, and no participant was counted twice. For the factorial trial, Zanetti et al. (55), the placebo-background stratum contributed to the Supporting inflammatory change-score analyses, with the alternative statin-background stratum and the merged trial examined in Sensitivity analyses; all fibrinogen contrasts remained Narrative. Companion reports of the same trial were merged under a single trial identifier before extraction, so no trial contributed more than once through duplicate publication.

### 2.7 Synthesis methods

Domain-level risk-of-bias judgments are provided in **Supplementary Table S3**; robustness analyses are reported in **Supplementary Table S4**; and subgroup eligibility, separate contrast-form analyses, and moderator screening are detailed in **Supplementary Table S5**. Randomized, quasi-randomized and non-randomized controlled studies were eligible together because the evidence base for this question is small. The consequences of that choice were handled analytically: risk of bias was assessed with the tool matched to each design, the design composition and statistical weight of non-randomized evidence were reported, and any mixed-design construct was additionally restricted to randomized trials. For inflammatory outcomes, only post-intervention final-value standardized effects entered the primary analysis; change-score standardized effects were analyzed separately as supporting evidence. A contrast-form stratum was meta-analyzed when at least two independent trials contributed, whereas a one-trial stratum was presented as an individual effect. Meta-analyses used inverse-variance random-effects models, restricted maximum likelihood estimation of between-study variance, and Hartung-Knapp-Sidik-Jonkman confidence intervals with an *ad hoc* variance correction (26, 27). Heterogeneity was summarized by τ^2^, I^2^, the Cochran Q test (28), and, when at least three trials contributed, a 95% prediction interval (29). Statistical analyses were performed in Python 3.12 using purpose-written scripts with NumPy 2.4.4 and SciPy 1.17.1 (30, 31).

The decision to separate standardized effects by contrast form and to designate final-value analyses as primary was made after data extraction and was not specified in the registered protocol; it is therefore a *post hoc* analytic decision. All subgroup, contrast-form, and robustness analyses are likewise *post hoc* exploratory. Final-value and change-score estimates were compared descriptively only. No formal between-form test was performed: contrast form was confounded with trial identity and other study characteristics across only one to four trials per stratum, so these data cannot establish an independent causal or statistical contribution of contrast form to heterogeneity. Subgroup analysis was restricted to clinically interpretable moderators with at least three trials in every level, a condition met only by CD4+ T-cell count, for which intervention type and overall risk of bias were examined. No meta-regression was fitted because the number of trials per construct was insufficient for a stable slope.

*Post hoc* robustness analyses repeated eligible meta-analyses after a single change to the analysis set or variance model: a common-effect model; exclusion of the trial with a recreationally active comparator; restriction to trials with directly reported standard deviations; and alternative handling of the factorial trial within the separate change-score evidence. For CD4+ T-cell count, additional analyses restricted the set to randomized trials, final-value contrasts, change-score contrasts, or trials other than the one with marked baseline imbalance, and recomputed one flagged dispersion as a standard error. Because final-value and change-score standardized effects were analyzed separately, neither was treated as a sensitivity analysis of a mixed-form reference estimate.

Publication bias was not assessed by funnel-plot methods for any construct, because fewer than 10 trials contributed to each, which is below the minimum recommended for those methods (23, 32). Small-study effects therefore cannot be excluded.

Dose-response synthesis was planned but not undertaken: no construct had two or more trials contributing three or more quantifiable exposure levels, so the prespecified entry condition was not met and all quantitative synthesis is pairwise.

### 2.8 Risk of bias and certainty of evidence

Risk of bias was assessed for each included trial with respect to the eligible biomarker and T-cell outcomes contributing to this review, using the tool matched to study design: RoB 2 for randomized and quasi-randomized trials (33), and ROBINS-I for the non-randomized controlled trial (34). Risk of bias was assessed by one reviewer (JZ), and every domain-level judgment together with its supporting reasoning was then checked against the source report by a second reviewer (ZL); queries raised at that check were resolved by discussion and by returning to the source report. The judgments were therefore not made independently in duplicate, and this is carried among the limitations in Section 4.5. Domain-level and overall judgments with their supporting reasoning are given in **Supplementary Table S3**.

Certainty of evidence was rated with GRADE at construct level across risk of bias, inconsistency, indirectness, imprecision and publication bias (35–38). Ratings for the six inflammatory constructs were based on the primary final-value evidence; CD4+ T-cell count was rated from the full unstandardized mean-difference analysis. The starting point was set from the design composition of each construct. The primary evidence for all six inflammatory constructs consists exclusively of randomized trials and therefore starts at high certainty. CD4+ T-cell count is the only mixed-design body: six randomized trials and one non-randomized trial, Gomes et al. (45), which carried 19.7% of the random-effects weight. That body also started at high certainty on the strength of the randomized evidence, while the serious ROBINS-I judgment for the non-randomized trial was reflected in the risk-of-bias domain and examined in a randomized-only analysis. No outcome was downgraded for indirectness solely because both groups received the same placebo, medication, or care background; balanced co-interventions preserve the direct exercise-versus-control contrast. The recreationally active comparator met the prespecified eligibility definition and its influence was examined in a robustness analysis. Domain-by-domain reasoning is reported in **Supplementary Table S6**.

## 3 Results

### 3.1 Study selection

The search of six databases to 1 June 2026 identified 1,158 records. After 645 duplicates were removed, 513 records were screened on title and abstract and 462 were excluded. Of the 51 reports assessed in full text, 33 were excluded, for wrong outcome (14), wrong intervention (12), wrong population (5) and wrong publication type (2). Eighteen reports met the eligibility criteria (18, 39–55). These 18 reports correspond to 15 trials, three of which were described in two companion reports each (43, 44, 47, 48, 53, 54). Thirteen trials contributed to at least one meta-analysis. Two trials were described narratively only: Khoramipour et al. (46), whose outcome values are available only as bar charts without numerical values or dispersion (46), and Yarasheski et al. (52), whose eligible outcome, adiponectin, was designated for narrative synthesis (52). Thirteen trials were randomized—12 individually randomized and one block-randomized—whereas one trial was quasi-randomized and one was non-randomized. No report required translation: one included report appears in a bilingual journal with a Portuguese title and abstract alongside an English full text (45), and the English full text was used for eligibility assessment and extraction. The flow of records and trials is shown in **Figure 1**.

**Figure 1:**
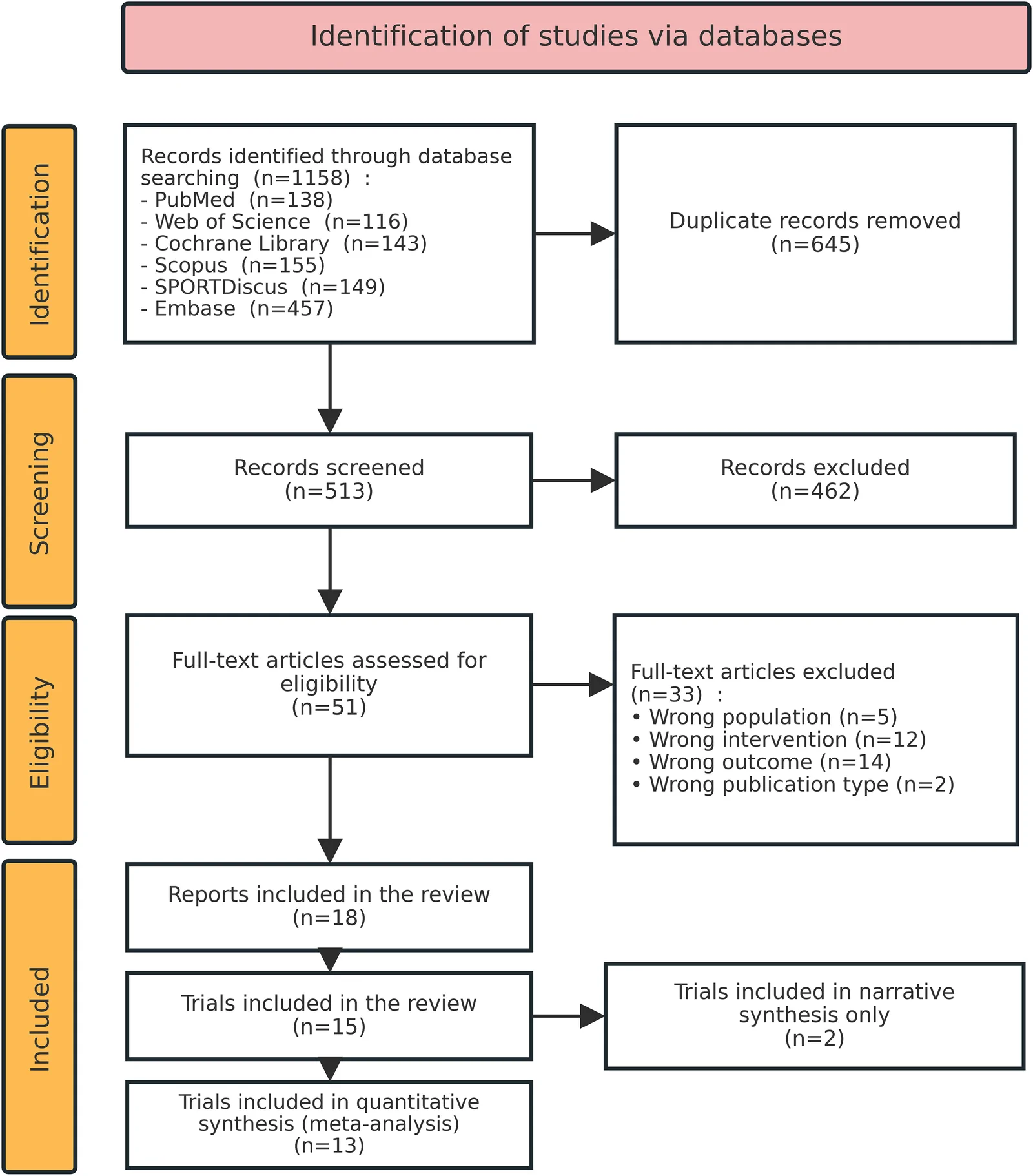
Flow diagram. Flow of records and of trials through the review, following the PRISMA 2020 statement. Databases were searched to 1 June 2026; no separate search of trial registries or other grey-literature sources was undertaken. Counts at the identification and screening stages are records or reports, as labeled; the included stage is resolved to the level of the trial. The 18 included reports correspond to 15 trials, of which 13 contributed to at least one meta-analysis and 2 were synthesized narratively only: Khoramipour et al. (46), whose outcome values are available solely as bar charts, and Yarasheski et al. (52), whose eligible adiponectin outcome was designated for narrative synthesis.

### 3.2 Characteristics of included trials

Trial characteristics are given in **Table 1** and intervention and comparator characteristics in **Table 2**. The 15 trials allocated 591 participants across the 14 trials reporting an allocation figure, and 456 participants contributed outcome data. Twelve were randomized controlled trials, one used block randomization, one was quasi-randomized by even/odd allocation, and one was a non-randomized controlled clinical trial. One trial used a 2×2 factorial design combining exercise with rosuvastatin or placebo, and one was a three-arm trial.

**Table 1:** Characteristics of the included trials.

Trial	Country	Design	Randomized/analyzed, n	Population and baseline status	Mean age, y	Female, %	Comparator	Follow-up, wk
Brito-Neto et al. (39)	Brazil	Randomized controlled trial	19/19	Clinically stable adults living with HIV/AIDS	39.2	42.1	Non-exercise control	12
Dianatinasab et al. (40)	Iran	Block-randomized controlled trial	40/30	Women aged 20–40 living with HIV	29.3	100	Non-exercise control	12
Dudgeon et al. (41)	United States	Randomized controlled trial	111/26	Men living with HIV without active opportunistic infection	44.5	0	Non-exercise control	6
Ghayomzadeh et al. (42)	Iran	Randomized controlled trial	21/18	Sedentary adults living with HIV	39.0	33.3	Non-exercise control	8
Ghayomzadeh et al. (43)	Iran	Randomized controlled trial; two companion reports	40/37	Adults aged 18–45 with stable HIV treatment	38.1	50	Non-exercise control	26.1
Gomes et al. (45)	Brazil	Non-randomized controlled clinical trial	NR/29	Adults living with HIV; no recent opportunistic disease	45.0	41.4	Non-exercise control	12
Khoramipour et al. (46)	Iran	Quasi-randomized controlled trial (even/odd allocation)	30/26	Adults with AIDS/HIV diagnosis ≥1 year	40.9	NR	Non-exercise control	12
Pedro et al. (47)	Brazil	Randomized controlled trial; two companion reports	58/28	Adults living with HIV; subgroup analyses by lipodystrophy phenotype	44.8	57.1	Recreational active control without structured resistance training	16
Piraux et al. (49)	Belgium	Pilot randomized controlled trial	25/17	Adults living with HIV without AIDS	46.7	35.3	Non-exercise control	6
Ryan et al. (50)	United States	Randomized controlled trial	33/28	Sedentary adults aged ≥50 years living with HIV; high cardiometabolic comorbidity burden	61.1	9.1	Non-exercise control	16
Soares et al. (51)	Brazil	Three-arm randomized controlled trial	41/41	Sedentary adults aged 18–60 living with HIV	39.5	51.2	Non-exercise control	12
Vingren et al. (18)	United States	Randomized controlled trial	16/16	Men with HIV receiving inpatient substance-abuse treatment	42.0	0	Non-exercise control	6
Yarasheski et al. (52)	United States	Randomized controlled trial	44/39	Insulin resistance/impaired glucose tolerance with central adiposity	45.0	12.8	Pioglitazone-only non-exercise control	17.4
Zanetti et al. (53)	Brazil	Randomized controlled trial; two companion reports	30/30	Adults living with HIV on stable HAART	41.1	43.3	Non-exercise control	12
Zanetti et al. (55)	Brazil	2×2 factorial randomized, double-blind, placebo-controlled trial	83/72	Adults with HIV and dyslipidemia, no prior statin use	41.9	48.6	Non-exercise control	12

Trials are listed alphabetically by first author. Eighteen reports correspond to 15 trials; companion reports of the same trial are merged under a single trial label. Randomized/analyzed gives the number allocated (or eligible, for the non-randomized trial) and the number contributing outcome data. Mean age is the pooled or all-participant mean reported by each trial.

ART, antiretroviral therapy; HAART, highly active antiretroviral therapy; HIV, human immunodeficiency virus; NR, not reported; n, number of participants; wk, weeks; y, years.

**Table 2:** Intervention and comparator characteristics of the included trials.

Trial	Intervention content	Weeks	Sessions/wk	Session, min	Resistance prescription	Aerobic component	Delivery and supervision	Comparator condition
Brito-Neto et al. (39)	Resistance training only	12	2	NR	Intensity: OMNI-RES 7–8; 3 sets × 8–10 repetitions; 7 exercises; Progressive load adjustment	5 min/session; Light cycle warm-up only	Supervised; Facility-based	No training program
Dianatinasab et al. (40)	Combined aerobic and resistance exercise	12	3	75–95	Intensity: 50–55% initial load, +5% every 2 weeks; 3 sets × 8 repetitions; 6 exercises; Stepwise progressive loading every 2 weeks	45 min/session; Cycling at 40–45% HRmax	Physiotherapist-supervised; Facility-based	Routine monitoring and consultation services
Dudgeon et al. (41)	Combined aerobic and circuit resistance exercise	6	2	NR	Intensity: Initial 60% of 3RM; adjusted to a 12-repetition load; circuit format; 12 repetitions; Load adjusted to maintain 12 repetitions	30 min/session; 60–75% age-predicted HRmax	Trained exercise physiologist; Facility-based; Eligibility for analysis required ≥10/12 sessions	Delayed/no intervention
Ghayomzadeh et al. (42)	Resistance training only using elastic bands/body weight	8	3	NR	Intensity: 12–15RM to volitional failure; 12–15 repetitions; Two-phase progressive elastic-band/body-weight circuit	None	Hospital-based supervised	Standard care
Ghayomzadeh et al. (43)	Combined resistance and aerobic training	26.1	3	NR	Intensity: 8-, 12-, and 20-repetition sessions with adjusted loads; 3 sets × 8/12/20 repetitions; 8 exercises; Reverse linear periodization in 2022 report; described as nonlinear in 2021 companion report	20 min/session; 65–80% HRmax after 5-min warm-up	Supervised; Facility-based; Adherence: Men 85.2%; women 89.4%	Maintain habitual lifestyle
Gomes et al. (45)	Combined aerobic, strength and flexibility exercise	12	3	90	Intensity: 60–80% of 12RM; 3 sets × 12 repetitions; 5 exercises; 12RM reassessed every 2 weeks	30 min/session; PWC150-based aerobic exercise	Supervised; Facility-based	Waiting list or low attendance
Khoramipour et al. (46)	Combined aerobic and resistance training	12	3	NR	Intensity: 40–65% 1RM; 2–4 sets × 12–20 repetitions; 4 exercises; Stepwise progression every 4 weeks; 1RM reassessed every 2 weeks	45 min/session; 40–65% HRmax; aerobic duration 30→45→60 min	Supervised; sessions around 17:00; Facility-based; Adherence: ≥90% required for analysis	No intervention
Pedro et al. (47)	Concurrent aerobic and resistance training	16	3	NR	Intensity: 8–12RM to mechanical failure; final phase 8–10RM; 2–3 sets × 8–12 repetitions; Major muscle groups; Progression from 2×10–12RM to 3×8–10RM across phases	15–20 min/session; 50–70% heart-rate reserve	Supervised; Facility-based	Recreational active control without structured resistance training
Piraux et al. (49)	Telerehabilitation-delivered combined aerobic and resistance training	6	3	75	Intensity: 60–80% estimated 1RM; 3 sets × 10 repetitions; 3 exercises; Prescribed multi-modal program; progression NR	50 min/session; Intervals at 60–80% HRmax plus steady modes	Remote program with weekly telephone support; public fitness center; Adherence: Retention 69%	No change in lifestyle
Ryan et al. (50)	Combined aerobic and resistance exercise	16	3	NR	Intensity: 50–65% 1RM initially; loads increased approximately 10%; 1–2 sets × 8→12 repetitions; 6 exercises; Progressive volume/intensity	45 min/session; 50% HRR progressing to 70–80% HRR	Supervised; Facility-based	Maintain sedentary behavior/standard care
Soares et al. (51)	Three-arm combined resistance and aerobic exercise trial	12	3	NR	Intensity: Mon 4–6RM; Wed 15–20RM; Fri 8–12RM; 3 sets × 4–20 repetitions; 7 exercises; Daily undulating periodization	Variable: intervals or 20 min; 90%, 75%, or 70% target HR by session	Individually supervised; Facility-based; Adherence: 88.4%	No structured exercise
Vingren et al. (18)	Resistance training added to inpatient standard care	6	3	NR	Intensity: 5–12RM zones across sessions; 3–5 sets × 5–12 repetitions; Multiple free-weight/cable exercises; Nonlinear/undulating 6-week schedule	None	Supervised; Inpatient facility	Inpatient standard care
Yarasheski et al. (52)	Combined exercise on a pioglitazone background	17.4	3	90–120	Intensity: 1–2 sets of 8→12 repetitions to fatigue; loads increased ~10%; 1–2 sets × 8–12 repetitions; 7 exercises; Progressive loading	50–85% heart-rate reserve	Supervised; Facility-based; Adherence: 92%	No structured exercise
Zanetti et al. (53)	Nonlinear resistance training only	12	3	NR	Intensity: 4–6RM/15–20RM/8–12RM by session; 3 sets × 4–20 repetitions; 6 exercises; Nonlinear/daily undulating	None	Supervised; Facility-based; Adherence: 100%	Usual daily activities
Zanetti et al. (55)	2×2 factorial: exercise training and rosuvastatin/placebo	12	3	NR	Intensity: 4–6RM/15–20RM/8–12RM by session; 3 sets × 4–20 repetitions; 6 exercises; Nonlinear resistance + polarized endurance	Session-specific: 7×30 s; 6×2 min; or 25 min; 85–90% HRR intervals; 70%/50% HRR intervals; 60% HRR steady	Supervised; Facility-based; Adherence: 91.6%	No structured exercise

Values are reproduced as prescribed in each report. Resistance intensity is given in the units used by the source trial, which differ across trials (percentage of one-repetition maximum, repetition-maximum zones, or ratings of perceived exertion) and are therefore not converted to a common scale. Trials that prescribed no aerobic component are shown as None.

1RM, one-repetition maximum; HRmax, maximum heart rate; min, minutes; NR, not reported; OMNI-RES, OMNI resistance exercise scale; PWC150, physical working capacity at a heart rate of 150 beats per minute; RM, repetition maximum; wk, week.

Participants were middle-aged adults, with trial mean ages from 29.3 to 61.1 years. The proportion of women ranged from 0% in the two trials confined to men to 100% in the trial restricted to women, and was not reported by one trial. Populations were mostly clinically stable adults on antiretroviral therapy, but included specific groups: men receiving inpatient substance-abuse treatment, adults aged 50 years or older with a high cardiometabolic comorbidity burden, adults with dyslipidemia and no prior statin use, and adults with insulin resistance and central adiposity.

Interventions ran from 6 to 26.1 weeks, most commonly 12 weeks, at two or three sessions per week. Four trials delivered resistance training alone; the remainder combined resistance work with an aerobic component. Resistance prescriptions were expressed on non-comparable scales — percentage of one-repetition maximum, repetition-maximum zones, or ratings of perceived exertion — and progression rules varied from fixed stepwise increments to reassessment every two weeks. Almost all programs were supervised and facility- or hospital-based; one was delivered as telerehabilitation. Where adherence was reported it was generally high, from 85.2% to 100%, although one trial reported 69% retention and two conditioned inclusion in the analysis on attendance thresholds. Comparators were most often no structured exercise or usual care; one trial used a recreationally active control, one a pioglitazone-only background, and one a shared placebo or statin background in both arms.

### 3.3 Risk of bias

Domain-level reasoning is given in **Supplementary Table S3**. No trial reached an overall low risk of bias. Of the 14 trials assessed with RoB 2, five were judged at high risk (40, 41, 46, 47, 49) and nine raised some concerns. The single non-randomized trial was rated at serious risk with ROBINS-I (45), driven by confounding from self-selected intervention uptake and by participant selection that depended partly on post-enrollment attendance and waiting-list status.

The dominant recurring problem was the randomization process: allocation concealment was undescribed in almost every report, and one trial used a predictable even/odd allocation (46). Missing outcome data drove several high-risk judgments, with 25% to more than 50% of allocated participants lacking analyzed post-intervention outcome data in three trials (40, 47, 49) and a biomarker subset of roughly a quarter of the randomized cohort in another (41). Two trials restricted analysis to participants completing a minimum number of sessions, producing per-protocol analyses that do not estimate the effect of assignment (41, 46). By contrast, measurement of the outcome was judged low risk throughout, since all biomarkers and cell counts were measured by standardized laboratory assays or flow cytometry, frequently with blinded assessors. Selection of the reported result raised some concerns in most trials, chiefly because no prospective outcome-specific analysis plan was accessible.

### 3.4 Inflammatory biomarkers

The primary results for the six inflammatory constructs are based on final-value contrasts. These results are reported in **Table 3** and displayed in the six-panel forest plot in **Figure 2**; per-construct primary forest plots are provided as **Supplementary Figures S1** to **S6**. Change-score evidence is reported separately as supporting evidence in **Supplementary Table S5** and **Figure 3**. Every inflammatory construct is contributed exclusively by randomized trials: neither the quasi-randomized trial (46) nor the non-randomized trial (45) contributes to these analyses.

**Table 3:** Primary effects of resistance training or resistance-containing combined exercise versus non-exercise control.

Outcome	Trials, k	Participants, n	Effect metric	Effect (95% CI)	P	I^2^, %	τ^2^	95% prediction interval
Interleukin-6 (IL-6)	4	98	SMD (final values)	−0.19 (−1.14 to 0.76)	0.571	48.5	0.15	−2.29 to 1.91
Tumor necrosis factor alpha (TNF-α)	3	72	SMD (final values)	0.07 (−1.44 to 1.57)	0.865	49.9	0.17	−6.75 to 6.89
C-reactive protein (CRP)	3	66	SMD (final values)	−0.02 (−1.46 to 1.41)	0.952	46.4	0.16	−6.58 to 6.54
Interleukin-1 beta (IL-1β)	2	42	SMD (final values)	−0.47 (−10.09 to 9.15)	0.646	81.4	0.94	Not estimable
Interleukin-8 (IL-8)	1	28	SMD (final value)	−0.63 (−1.39 to 0.12)	0.100	—	—	—
Interleukin-10 (IL-10)	2	44	SMD (final values)	0.04 (−11.37 to 11.46)	0.969	87.4	1.41	Not estimable
CD4+ T-cell count	7	184	MD (cells/mm^3^)	124.2 (54.6 to 193.7)	0.005	71.3	2653	−27.0 to 275.4

For the six inflammatory constructs, final-value standardized mean differences are the primary analyses and change-score standardized mean differences are reported separately in **Supplementary Table S5** and **Figure 3**. Random-effects meta-analysis was used when at least two trials shared the final-value form. The interleukin-8 row is an individual study result and is not a pooled estimate. CD4+ T-cell count is an unstandardized mean difference in cells/mm^3^, for which final-value and change-score contrasts retain the same absolute unit and were combined. Random-effects models used restricted maximum likelihood estimation of τ^2^ and Hartung-Knapp-Sidik-Jonkman confidence intervals. Prediction intervals were estimated only when at least three trials contributed.

P is the p value for the pooled effect estimate, except for interleukin-8, where it is the p value for the individual study effect. The p value shown beneath pooled forest-plot panels is the Cochran Q test for heterogeneity and is labeled p(Q).

No dose-response synthesis was undertaken. No construct had at least two trials contributing three or more quantifiable exposure levels, so all quantitative synthesis is pairwise.

The additional outcomes designated for Narrative synthesis are listed in **Supplementary Table S7**; their reporting is distinct from one-trial Primary or Supporting results.

CI, confidence interval; CRP, C-reactive protein; I^2^, percentage of variability across trials attributable to heterogeneity rather than chance; IL, interleukin; k, number of trials; MD, mean difference; n, number of participants; P, p value for the effect estimate; SMD, standardized mean difference; TNF-α, tumor necrosis factor alpha; τ^2^, estimated between-trial variance.

**Figure 2:**
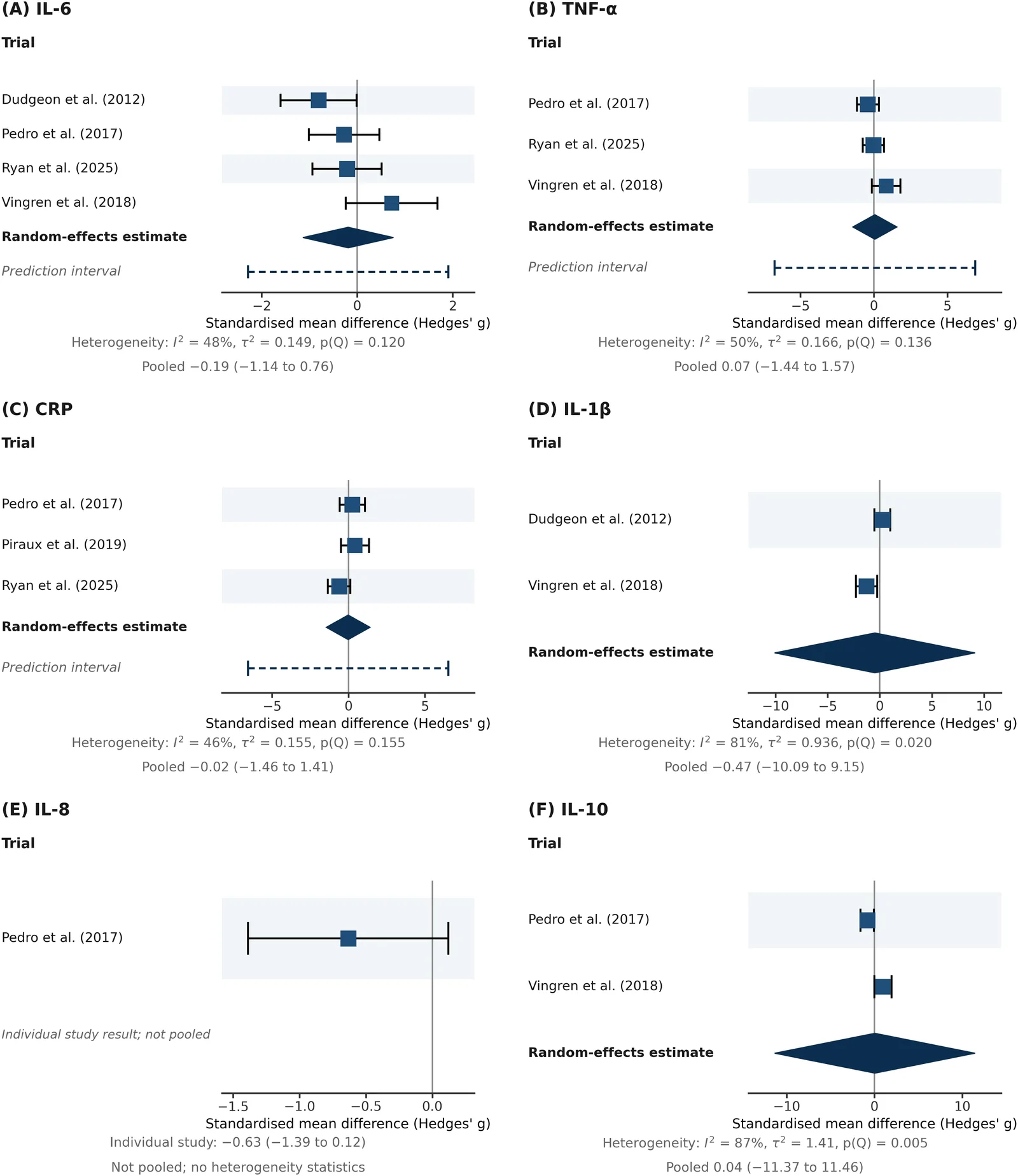
Primary final-value analyses of the six inflammatory biomarker constructs: **(A)** interleukin-6 (IL-6), **(B)** tumor necrosis factor alpha (TNF-α), **(C)** C-reactive protein (CRP), **(D)** interleukin-1 beta (IL-1β), **(E)** interleukin-8 (IL-8), and **(F)** interleukin-10 (IL-10). Standardized mean differences (Hedges’ g) are shown as exercise minus comparator with 95% confidence intervals. Squares are trial estimates with area proportional to random-effects weight; diamonds are pooled estimates. Every panel is restricted to final-value contrasts and reproduces the analyses reported in **Table 3** and in **Supplementary Figures S1** to **S6**; change-score contrasts do not appear in this figure and are reported separately in **Figure 3**. Interleukin-8 is represented by one final-value trial and is displayed as an individual study result without a pooled diamond. Dashed horizontal lines are 95% prediction intervals, which are shown only for meta-analyses with at least three trials. Negative values indicate lower concentrations in the exercise group for the pro-inflammatory constructs; the clinical direction of interleukin-10 is context dependent. The horizontal scale is set independently in each panel to the range of the estimates it displays, so scales are not comparable between panels. CI, confidence interval; IL, interleukin; p(Q), p value of the Cochran Q test for heterogeneity; SMD, standardized mean difference; TNF-α, tumor necrosis factor alpha; τ^2^, between-study variance.

**Figure 3:**
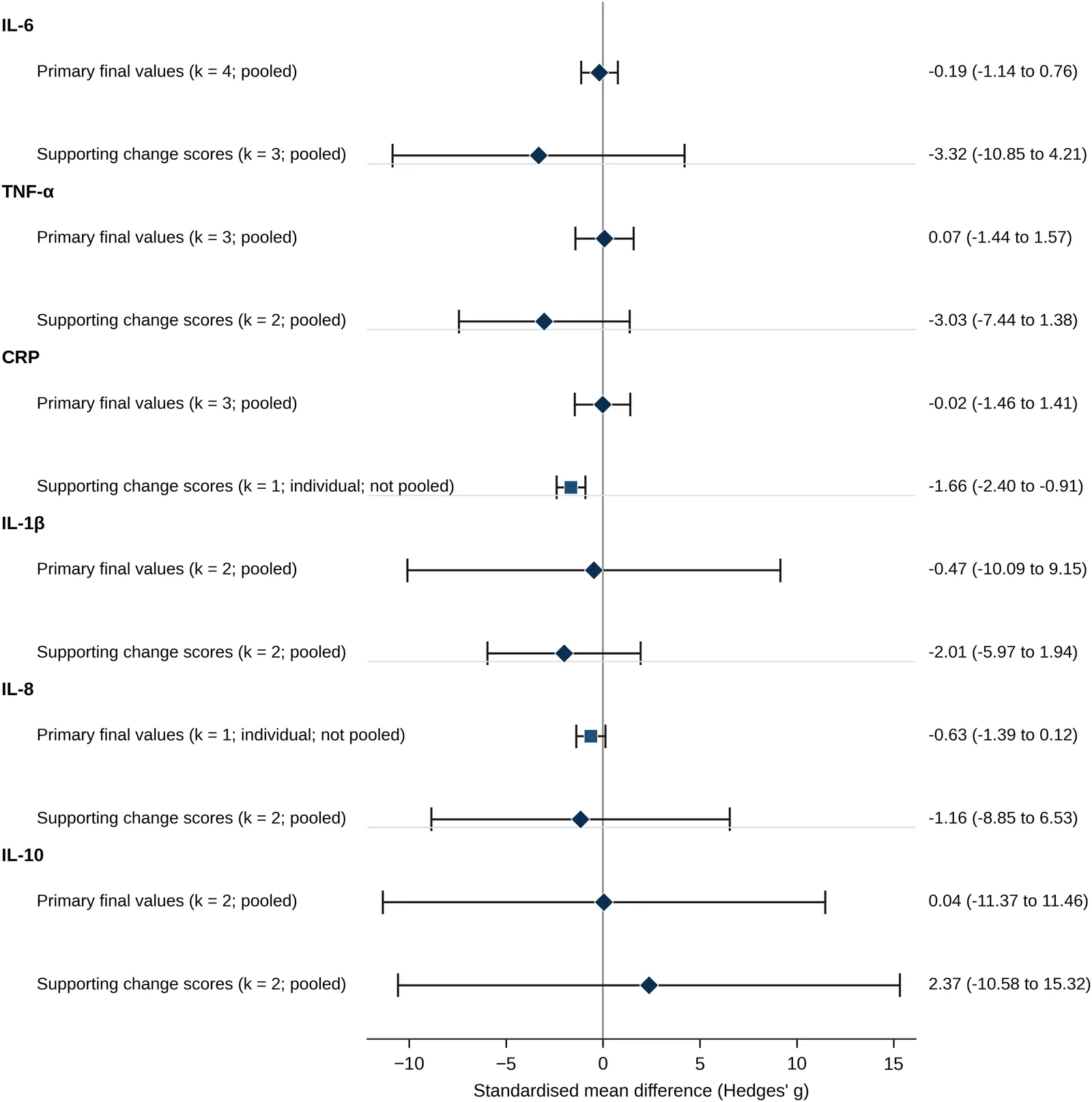
Separate final-value and change-score standardized effects for the inflammatory constructs. Final-value standardized mean differences are the primary analyses and change-score standardized mean differences are supporting analyses. Diamonds denote random-effects meta-analyses with at least two trials; squares denote one-trial individual results that were not pooled. The two contrast forms use different standardizing standard deviations and are therefore presented separately. Differences between rows are descriptive only: contrast form is confounded with trial characteristics across only one to four trials per stratum, so no independent causal or statistical contribution of contrast form to heterogeneity can be established. k, number of trials; CI, confidence interval.

For interleukin-6, four final-value trials with 98 participants yielded a pooled standardized mean difference of −0.19 (95% CI −1.14 to 0.76), with I^2^ = 48%, τ^2^ = 0.15 and a prediction interval from −2.29 to 1.91 (**Figure 2A**; **Supplementary Figure S1**). For tumor necrosis factor alpha, three trials with 72 participants gave 0.07 (−1.44 to 1.57), with I^2^ = 50% and a prediction interval from −6.75 to 6.89 (**Figure 2B**; **Supplementary Figure S2**). For C-reactive protein, three trials with 66 participants gave −0.02 (−1.46 to 1.41), with I^2^ = 46% and a prediction interval from −6.58 to 6.54 (**Figure 2C**; **Supplementary Figure S3**). For interleukin-1 beta, two trials with 42 participants gave −0.47 (−10.09 to 9.15), with I^2^ = 81% (**Figure 2D**; **Supplementary Figure S4**). The final-value evidence for interleukin-8 consisted of one trial with 28 participants, −0.63 (−1.39 to 0.12), and was reported as an individual study result rather than pooled (**Figure 2E**; **Supplementary Figure S5**). For interleukin-10, two trials with 44 participants gave 0.04 (−11.37 to 11.46), with I^2^ = 87% (**Figure 2F**; **Supplementary Figure S6**). Prediction intervals were not estimated for the two-trial meta-analyses.

Every primary final-value confidence interval included no effect. Change-score standardized effects were synthesized separately. Their pooled confidence intervals also included no effect for interleukin-6, tumor necrosis factor alpha, interleukin-1 beta, interleukin-8 and interleukin-10. C-reactive protein was represented by one change-score trial, −1.66 (−2.40 to −0.91); this individual result favored exercise but was not a meta-analysis. Thus, neither contrast-form evidence set provided stable construct-level evidence of an inflammatory effect.

### 3.5 CD4+ T-cell count

Seven trials with 184 participants contributed to CD4+ T-cell count (**Figure 4**). Six were randomized and one was non-randomized (45); the non-randomized trial carried 19.7% of the random-effects weight, and restricting the analysis to randomized trials gave 114.0 cells/mm^3^ (28.2 to 199.8; six trials, I^2^ = 66%). This is the only construct to which more than one study design contributes. Because CD4+ effects were unstandardized mean differences in the same absolute unit, final-value and change-score contrasts could be combined without the scale-comparability problem that applies to standardized effects. The pooled mean difference was 124.2 cells/mm^3^ (95% CI 54.6 to 193.7; p = 0.005), with I^2^ = 71%, τ^2^ = 2,653 and a prediction interval from −27.0 to 275.4. Six of the seven trial estimates were above zero. The confidence interval suggests a possible increase, but the prediction interval includes no effect, so a future trial in a new setting could plausibly observe no difference.

**Figure 4:**
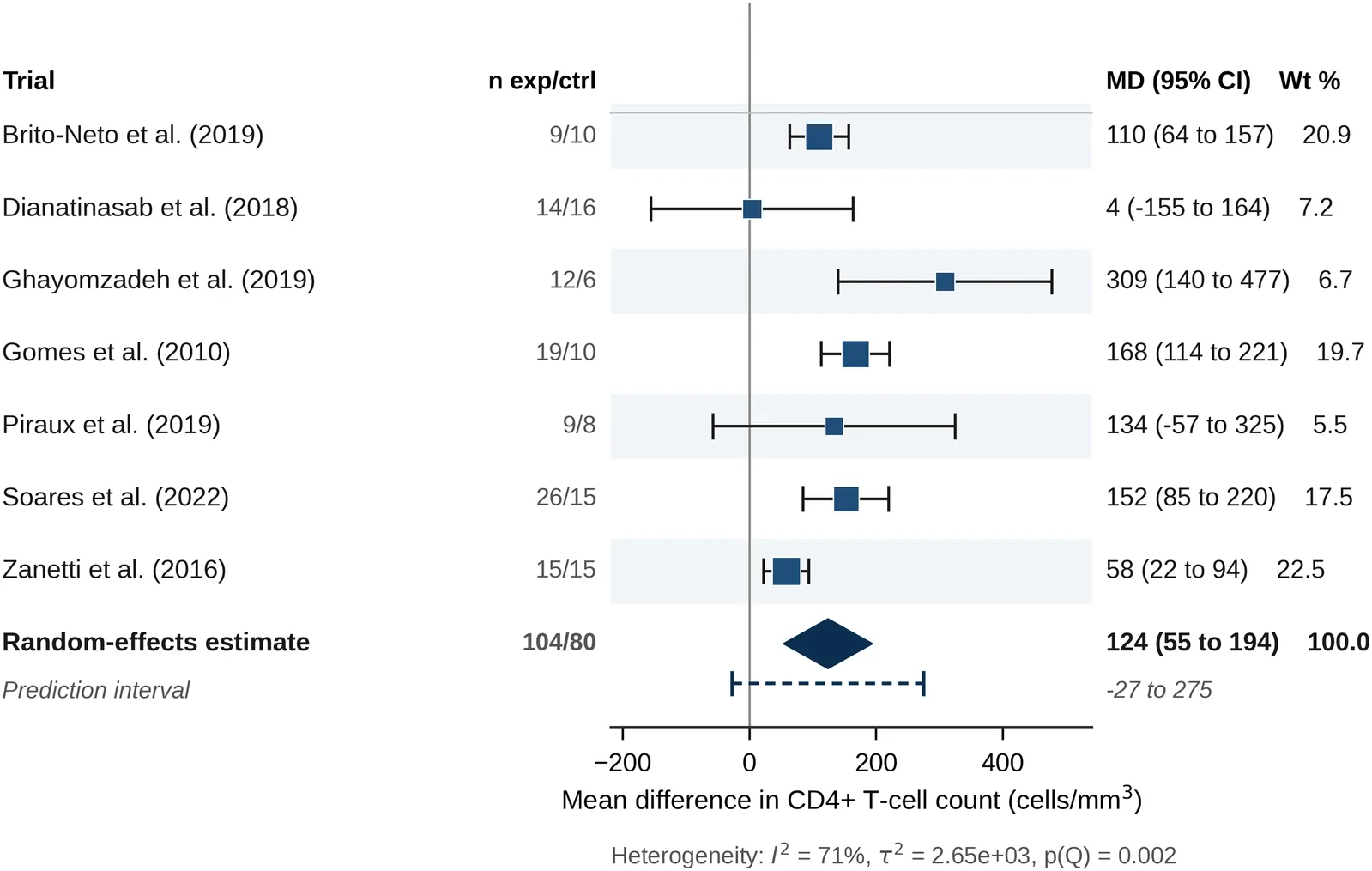
Forest plot of CD4+ T-cell count. The Primary CD4+ analysis combines final-value and change-score mean differences in cells/mm^3^, oriented as exercise minus comparator with 95% confidence intervals; squares are trial estimates with area proportional to the random-effects weight, the diamond is the pooled estimate and the dashed line beneath it is the 95% prediction interval. Pooling used an inverse-variance random-effects model with restricted maximum likelihood estimation of the between-study variance and a Hartung-Knapp-Sidik-Jonkman confidence interval. n exp/ctrl, number analyzed in the exercise and comparator groups; Wt, weight. CI, confidence interval; I^2^, percentage of variability attributable to heterogeneity rather than chance; MD, mean difference; p(Q), p value of the Cochran Q test for heterogeneity, which is distinct from the p value for the pooled effect estimate reported in **Table 3**; τ^2^, between-study variance.

### 3.6 Heterogeneity, contrast form and subgroups

Separate analyses by contrast form are displayed in **Figure 3** and detailed in **Supplementary Table S5**. Change-score point estimates were farther from zero than final-value point estimates for all six inflammatory constructs; this is a descriptive comparison of estimates standardized using different dispersions and contributed by different trials. For example, interleukin-6 was −0.19 (−1.14 to 0.76; four final-value trials) and −3.32 (−10.85 to 4.21; three change-score trials), while tumor necrosis factor alpha was 0.07 (−1.44 to 1.57; three final-value trials) and −3.03 (−7.44 to 1.38; two change-score trials). Contrast form is confounded with trial identity, comparator condition, intervention characteristics, and dispersion reconstruction across only one to four trials per stratum. These data cannot establish an independent causal or statistical contribution of contrast form to heterogeneity, and no formal between-form inference was made. For CD4+ T-cell count, the separate final-value and change-score estimates were 140.5 and 100.8 cells/mm^3^, respectively, but the latter was based on only two trials and was very imprecise.

Subgroup analyses were possible only for CD4+ T-cell count (**Supplementary Figure S7**) and are *post hoc* exploratory. Neither intervention type (resistance training alone, 135.8 cells/mm^3^, −160.5 to 432.1, three trials; resistance-containing combined training, 150.7, 79.6 to 221.8, four trials) nor overall risk of bias (high or serious, 121.0, −97.2 to 339.2, three trials; some concerns, 130.1, −10.6 to 270.7, four trials) showed evidence of explaining between-trial variation. Programs also differed in modality, duration, intensity prescription and timing of blood sampling, but the evidence base was too small and these features too interdependent for formal testing. Comparators ranged from no structured exercise to a recreationally active control and balanced pharmacological backgrounds. A placebo or medication background applied equally to both groups preserves a direct exercise contrast; variation in comparator activity may nevertheless contribute to heterogeneity. Excluding the recreationally active comparator did not materially change the primary inference. These features support reading the estimates as summaries of a sparse and heterogeneous literature rather than as a single common effect.

### 3.7 Sensitivity analyses

Robustness analyses are reported in **Supplementary Table S4** and displayed in **Supplementary Figure S8**. For CD4+ T-cell count, the direction and approximate magnitude of the estimate survived every check: restricting to randomized trials gave 114.0 cells/mm^3^ (28.2 to 199.8); excluding the trial with a baseline CD4 difference of about 167 cells/mm^3^ in favor of the exercise arm gave 111.2 (50.9 to 171.6); treating one trial’s flagged dispersion as a standard error gave 116.4 (44.3 to 188.6); and restricting to final-value contrasts gave 140.5 (34.7 to 246.3). The two-trial change-score subset was very imprecise, at 100.8 (−495.5 to 697.2).

For the primary final-value inflammatory analyses, switching to a common-effect model did not produce an interval excluding no effect for any pooled construct. Excluding the recreationally active comparator or restricting to directly reported standard deviations also did not yield a stable pattern across constructs. Some reduced subsets contained only one trial and are therefore displayed as individual results, not pooled estimates. The separately synthesized change-score evidence and alternative handling of the factorial trial likewise remained imprecise. Three one-trial estimates in **Supplementary Table S4** had confidence intervals excluding no effect: the Supporting C-reactive protein change-score contrast, its merged-medication-strata Sensitivity contrast from the same factorial trial, and the interleukin-1 beta analysis restricted to a directly reported standard deviation. The two C-reactive protein contrasts are alternative representations of one trial, not independent replications; none of these one-trial results establishes a reliable construct-level effect.

Overall risk-of-bias judgments are summarized in **Table 4** and displayed in **Figure 5**; contrast-form estimates are displayed in **Figure 3**.

**Table 4:** Overall risk of bias of the included trials.

Trial	Tool	Overall judgment	Domains driving the overall judgment
Brito-Neto et al. (39)	RoB 2	Some concerns	D1 (randomization process): random allocation was stated, but sequence generation and allocation concealment were not reported. D5 (selection of the reported result): no prospective protocol or outcome-specific analysis plan was available and several analytical choices were possible.
Dianatinasab et al. (40)	RoB 2	High	D2 (deviations from intended interventions): participants were excluded after assignment for non-completion of exercise, so the complete-case analysis did not estimate the effect of assignment. D3 (missing outcome data): 25% of randomized participants lacked post-intervention data, with reasons plausibly related to intervention participation or outcome status.
Dudgeon et al. (41)	RoB 2	High	D2 (deviations from intended interventions): inclusion in the final analysis required completion of at least 10 of 12 sessions, creating a per-protocol analysis that excluded deviations after assignment. D3 (missing outcome data): outcome data were available for only a small and inconsistently reported subset of the 111 randomized participants; the biomarker analysis was approximately one quarter of the randomized cohort.
Ghayomzadeh et al. (42)	RoB 2	Some concerns	D1 (randomization process): random assignment was stated, but the sequence method and concealment were not reported. D3 (missing outcome data): 3/21 participants (14%) were missing and reasons were not reported; the balanced direction reduces but does not remove concern.
Ghayomzadeh et al. (43)	RoB 2	Some concerns	D1 (randomization process): equal random allocation was reported, but sequence generation and allocation concealment were not described.
Gomes et al. (45)	ROBINS-I	Serious	D1 (confounding): intervention uptake was self-selected and no adequate adjustment for prognostic differences was performed. D2 (selection of participants): control membership depended partly on post-enrollment attendance/dropout and waiting-list status, both related to intervention participation.
Khoramipour et al. (46)	RoB 2	High	D1 (randomization process): even/odd allocation was predictable and therefore did not provide concealed random allocation. D2 (deviations from intended interventions): the analysis excluded participants after assignment according to attendance, so it did not estimate the effect of assignment.
Pedro et al. (47)	RoB 2	High	D3 (missing outcome data): more than half of randomized participants lacked analyzed cytokine data, and the companion report used an even smaller biomarker subset with incomplete reconciliation of denominators and reasons.
Piraux et al. (49)	RoB 2	High	D3 (missing outcome data): 8/25 participants (32%) lacked outcome data, and reasons such as deterioration, motivation and unknown causes could relate to true outcomes.
Ryan et al. (50)	RoB 2	Some concerns	D1 (randomization process): 1:1 random allocation was stated, but sequence generation and allocation concealment were not reported. D3 (missing outcome data): 5/33 participants (15%) lacked final data and outcome-specific reasons were not fully reported in text.
Soares et al. (51)	RoB 2	Some concerns	D1 (randomization process): a computerized sequence was used, but allocation concealment was not reported.
Vingren et al. (18)	RoB 2	Some concerns	D1 (randomization process): random assignment was stated, but sequence generation and allocation concealment were not described. D5 (selection of the reported result): no prospective outcome-specific analysis plan was available for the broad cytokine panel and multiple analyses were possible.
Yarasheski et al. (52)	RoB 2	Some concerns	D1 (randomization process): random assignment was reported, but sequence generation and allocation concealment were not described. D3 (missing outcome data): 5/44 participants (11%) lacked final data and several losses were related to noncompliance, which could be prognostic.
Zanetti et al. (53)	RoB 2	Some concerns	D1 (randomization process): random allocation was stated, but the sequence method and concealment were not described.
Zanetti et al. (55)	RoB 2	Some concerns	D1 (randomization process): computerized randomization and blinding procedures were reported, but concealment at enrollment was not explicitly described. D3 (missing outcome data): 11/83 participants (13.25%) were missing, including five withdrawals without reasons; this could plausibly bias a small factorial contrast.

Judgments are reported as text only. The full study-by-domain grid is presented graphically and is not duplicated here; domain-level judgments with their supporting reasoning are given in **Supplementary Table S3**.

The overall judgment is the least favorable domain judgment, following the algorithm of each tool. No trial reached an overall low-risk judgment.

D1-D7, risk-of-bias domains as defined by the tool applied; RoB 2, revised Cochrane risk-of-bias tool for randomized trials; ROBINS-I, Risk Of Bias In Non-randomized Studies of Interventions.

**Figure 5:**
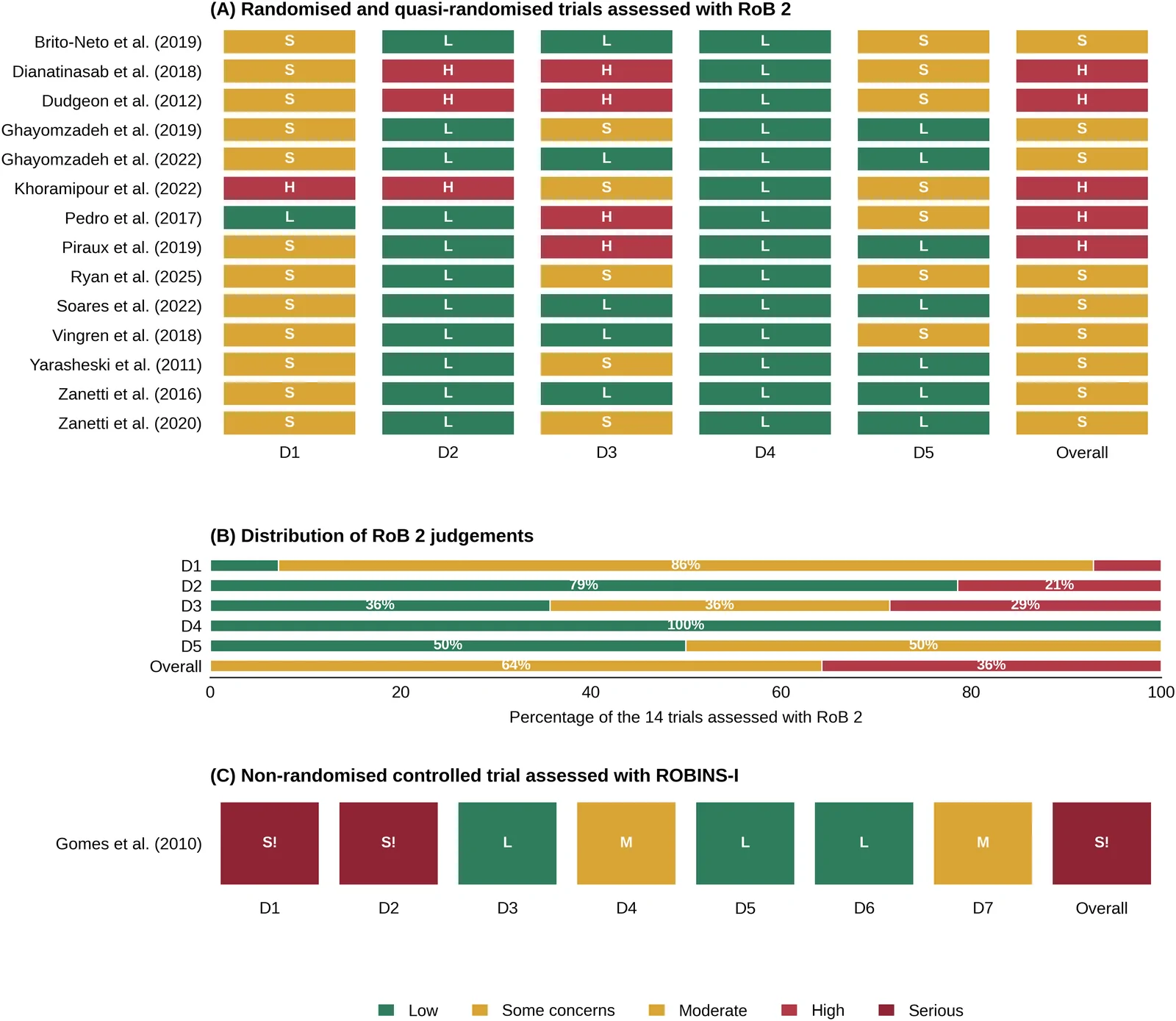
Risk of bias in the included trials. **(A)** Judgment for each randomized or quasi-randomized trial and each RoB 2 domain: D1, randomization process; D2, deviations from intended interventions; D3, missing outcome data; D4, measurement of the outcome; D5, selection of the reported result. The letter inside each cell repeats the judgment so that color is not the only encoding: L, low; S, some concerns; H, high. **(B)** Percentage of those trials receiving each judgment, by domain. **(C)** The single non-randomized controlled trial, assessed with ROBINS-I: D1, confounding; D2, selection of participants; D3, classification of interventions; D4, deviations from intended interventions; D5, missing data; D6, measurement of outcomes; D7, selection of the reported result; M, moderate; S!, serious. The two tools use different domains, so the ROBINS-I assessment is shown separately rather than merged into the same grid. RoB, risk of bias.

### 3.8 Outcomes not pooled

Twenty-three constructs were reported narratively, listed with reasons in **Supplementary Table S7**; the availability of outcome data across trials and constructs is displayed in **Supplementary Figure S9**. Most were reported by only one or two trials, including interferon gamma, interleukin-2, interleukin-4, interleukin-5, interleukin-18, granulocyte-macrophage colony-stimulating factor, soluble tumor necrosis factor receptor 2, vascular cell adhesion molecule 1, fibrinogen, D-dimer, adiponectin, leptin, irisin, myostatin, insulin-like growth factor 1, insulin-like growth factor-binding protein 3, growth hormone, testosterone, cortisol, CD8+ T-cell count and CD4+ T-cell percentage.

HIV-1 viral load was prespecified for narrative synthesis and reported by four trials (46, 49, 51, 53). Every contributing trial reported it as undetectable or below the assay limit, so no continuous contrast exists and no trial reported a deterioration in virological control during exercise. Brain-derived neurotrophic factor was reported by one trial in figure form only (46).

Adherence, where reported, was high (**Table 2**), but was documented by too few trials in a comparable format to support any quantitative comparison. No trial reported an increase in adverse events attributable to the exercise intervention.

### 3.9 Certainty of evidence

The summary of findings is given in **Table 5**, with domain-level reasoning in **Supplementary Table S6**. Certainty was very low for all seven constructs. The primary final-value inflammatory evidence comprised one to four trials and 28 to 98 participants per construct; CD4+ T-cell count comprised seven trials and 184 participants. Every construct was downgraded for risk of bias and imprecision. Interleukin-6, tumor necrosis factor alpha, C-reactive protein, interleukin-1 beta, interleukin-10 and CD4+ T-cell count were also downgraded for inconsistency. Inconsistency could not be assessed for interleukin-8 because only one final-value trial contributed, and imprecision was downgraded by two levels instead. No construct was downgraded for indirectness: all comparators met the prespecified target contrast, and placebo, medication or care backgrounds were applied equally to both groups. Publication bias was not assessed by funnel-plot methods because fewer than 10 trials contributed to every construct, so small-study effects cannot be excluded.

**Table 5:** Summary of findings: resistance training or resistance-containing combined exercise compared with non-exercise control in adults living with HIV.

Outcome	Trials (participants)	Anticipated effect (95% CI)	Certainty of the evidence	Reasons for the rating and interpretation
Interleukin-6 (IL-6)	4 (98)	−0.19 (−1.14 to 0.76) [SMD; final values]	Very low	Downgraded for risk of bias, inconsistency, and imprecision. The final-value estimate is compatible with an appreciable reduction and with no effect; no reliable conclusion can be drawn.
Tumor necrosis factor alpha (TNF-α)	3 (72)	0.07 (−1.44 to 1.57) [SMD; final values]	Very low	Downgraded for risk of bias, inconsistency, and imprecision. The final-value estimate is compatible with effects in both directions; no reliable conclusion can be drawn.
C-reactive protein (CRP)	3 (66)	−0.02 (−1.46 to 1.41) [SMD; final values]	Very low	Downgraded for risk of bias, inconsistency, and imprecision. The final-value estimate is close to no difference and is compatible with effects in both directions.
Interleukin-1 beta (IL-1β)	2 (42)	−0.47 (−10.09 to 9.15) [SMD; final values]	Very low	Downgraded for risk of bias, inconsistency, and imprecision. The two-trial estimate is extremely imprecise and supports no reliable conclusion.
Interleukin-8 (IL-8)	1 (28)	−0.63 (−1.39 to 0.12) [individual SMD; final value]	Very low	Downgraded for risk of bias and very serious imprecision. Between-trial inconsistency cannot be assessed from one trial; this is an individual study result, not a pooled estimate.
Interleukin-10 (IL-10)	2 (44)	0.04 (−11.37 to 11.46) [SMD; final values]	Very low	Downgraded for risk of bias, inconsistency, and imprecision. The two-trial estimate is compatible with large effects in either direction, and the direction of benefit is context dependent.
CD4+ T-cell count	7 (184)	124.2 (54.6 to 193.7) [MD, cells/mm^3^]	Very low	Downgraded for risk of bias, inconsistency, and imprecision. The confidence interval suggests a possible increase, but confidence is very limited because no contributing trial was at low risk of bias, heterogeneity is substantial, and the prediction interval crosses no effect.

Certainty was assessed with GRADE at construct level. Ratings for the six inflammatory constructs are based on the primary final-value evidence. These bodies consist exclusively of randomized trials and start at high certainty. CD4+ T-cell count is the only mixed-design body, comprising six randomized trials and one non-randomized trial carrying 19.7% of the random-effects weight; it starts at high certainty on the strength of the randomized evidence, while the serious ROBINS-I judgment is reflected in the risk-of-bias domain. No construct was downgraded for indirectness. A placebo, medication, or care background applied equally to both arms preserves the direct exercise contrast; the recreationally active comparator met eligibility and was examined in a *post hoc* robustness analysis. Publication bias was not assessed by funnel-plot methods because fewer than 10 trials contributed to every construct.

A negative standardized mean difference favors exercise for every inflammatory biomarker except interleukin-10, for which the direction of benefit is not defined; a positive mean difference favors exercise for CD4+ T-cell count.

CI, confidence interval; CRP, C-reactive protein; GRADE, Grading of Recommendations Assessment, Development and Evaluation; HIV, human immunodeficiency virus; IL, interleukin; MD, mean difference; SMD, standardized mean difference; TNF-α, tumor necrosis factor alpha.

## 4 Discussion

### 4.1 Principal findings

This review synthesized 18 reports of 15 trials, of which 13 contributed quantitative data, to determine whether resistance training or resistance-containing combined exercise alters inflammatory biomarkers or T-cell counts in adults living with HIV. The central finding is that the question remains unresolved. In the primary final-value analyses, the confidence intervals for all five pooled inflammatory constructs included no effect, and the interleukin-8 final-value evidence consisted of one inconclusive trial. Separate change-score analyses were also generally inconclusive. The pooled estimate for CD4+ T-cell count, 124.2 cells/mm^3^ (54.6 to 193.7), is the only construct-level confidence interval that excludes no effect and it was directionally stable across robustness analyses, but it rests on very-low-certainty evidence from seven small trials, none at low risk of bias, and its prediction interval crosses zero.

A biologically informed reading also cautions against expecting a large signal. The anti-inflammatory action of exercise is partly acute and transient, mediated by the cytokine response to each bout of contraction, and resting concentrations measured days after the final session may not capture it (56, 57). Interleukin-6 in particular behaves differently as an acutely released myokine than as a chronic inflammatory marker, which complicates its interpretation as a training outcome (57). More broadly, the relationship between exercise and immune function is regulatory rather than simply suppressive or stimulatory (58–60), so a uniform downward shift in every pro-inflammatory analyte was never the only plausible result.

Reporting these results as an absence of demonstrated effect rather than as demonstrated absence of effect matters. The primary inflammatory analyses contain only one to four trials and 28 to 98 participants per construct; among the pooled final-value analyses, I^2^ ranges from 46% to 87%. This evidence base has limited capacity to distinguish a moderate effect from none (61, 62). The appropriate reading of a wide interval centered away from the null is that the data are uninformative, not that the intervention is inert (63).

### 4.2 Contrast form and the interpretation of heterogeneity

The separate contrast-form analyses provide a methodological caution rather than an explanation of heterogeneity. The numerical differences between final-value and change-score estimates, and the differences in I^2^ between their separate analyses, are descriptive observations. Different standardizing dispersions provide a methodological reason to keep the SMD forms separate, not evidence that contrast form explains the observed heterogeneity. Because the strata contain different trials, contrast form is confounded with trial identity, comparator condition, intervention characteristics, and dispersion reconstruction, with only one to four trials per stratum. These data therefore cannot establish an independent causal or statistical contribution of contrast form to heterogeneity.

The practical implication is that final-value and change-score standardized effects should not be combined as though they share one scale. Reporting them separately prevents differences in standardization from being mistaken for a single construct-level effect, while avoiding the stronger and unsupported claim that reporting form explains the clinical variation. CD4+ T-cell count is different because it is expressed as an unstandardized mean difference in cells/mm^3^; final-value and change-score effects retain the same absolute unit. Future trials should report both forms with complete dispersion data and, ideally, the pre-post correlation, so either form can be synthesized consistently.

### 4.3 Relation to previous evidence

Earlier Cochrane-protocol reviews of aerobic exercise and of progressive resistive exercise in HIV concluded that these interventions are safe and can improve cardiorespiratory fitness, strength and body composition, while finding immunological outcomes considerably less clear (12, 13). Our results are consistent with that pattern and sharpen it: for the immunological and inflammatory endpoints specifically, the evidence remains too sparse and too heterogeneous to support conclusions, even a decade later and with the analysis restricted to resistance-containing interventions and organized by construct.

Some previous syntheses have reported favorable pooled effects of exercise on inflammatory markers in this population (16, 17). Our more conservative conclusions follow from organizing analyses by individual construct, separating final-value and change-score standardized effects, using Hartung-Knapp-Sidik-Jonkman intervals when trials are few (27), and reporting prediction intervals when estimable (29). In the primary final-value analyses, common-effect estimates also remained compatible with no effect for every pooled inflammatory construct, while the very wide random-effects intervals for two-trial syntheses show how little information is available about between-trial variation. The data therefore do not support a stable inflammatory effect under either variance model.

### 4.4 Interpretation and clinical implications

These findings should not be read as an argument against exercise for adults living with HIV. The rationale for physical activity in this population rests on well-established benefits for cardiorespiratory fitness, muscle mass and strength, body composition and psychological wellbeing (12–14), and on general population physical-activity guidance (64). Physical activity levels in this population are low (19), and no trial in this review reported a deterioration in virological control or an increase in adverse events attributable to exercise, with viral load remaining undetectable or below the assay limit wherever it was measured. Adherence, where reported, was high.

What these findings do establish is that the specific claim that resistance-containing exercise measurably lowers systemic inflammation in HIV is not currently supported at a level of certainty that would justify prescribing exercise for that purpose, or using biomarker change as a surrogate endpoint for exercise programs. That distinction carries weight because inflammatory markers are prognostically informative in their own right (65), and because lowering them pharmacologically has been shown to alter clinical outcomes in other settings (66), which makes an unsupported claim of biomarker modification more consequential than a merely academic overstatement. The CD4+ T-cell finding is the only primary pooled estimate whose confidence interval excludes no effect, but at very low certainty, with substantial heterogeneity and with a prediction interval that crosses no effect, it is a signal to be tested rather than a benefit to be relied upon; it should not be presented to patients or in guidelines as an established effect of exercise on immune reconstitution.

### 4.5 Strengths and limitations

Strengths include synthesis organized by clinical-biological construct rather than by outcome label; separation of final-value and change-score standardized effects rather than combining non-comparable standardizations; use of one effect metric within each analysis; direction harmonization; duplicate independent screening, full-text assessment and data extraction with third-reviewer adjudication, followed by source verification; explicit handling of multi-arm, factorial and multi-report trials so that no participant group contributed twice; and *post hoc* robustness analyses reported regardless of direction.

Several limitations qualify the conclusions. Risk of bias was assessed by one reviewer, with every judgment checked against the source report by a second reviewer rather than made independently in duplicate. No contributing trial was at overall low risk, and allocation concealment, missing outcome data, per-protocol exclusions and absent prospective analysis plans were recurrent concerns. The primary inflammatory evidence is extremely sparse: one to four trials and 28 to 98 participants per construct, with prediction intervals estimable for only three constructs. Separating contrast forms avoids combining differently standardized estimates but further reduces the number of trials in each analysis; with contrast form confounded with trial characteristics, neither its independent causal contribution nor its independent statistical contribution to heterogeneity can be established. Comparator activity, intervention modality, duration, intensity, and blood-sampling timing were incompletely described and could not be disentangled. Publication bias could not be assessed by funnel-plot methods because every construct had fewer than 10 trials. The search did not directly examine trial registries or other grey-literature sources, authors were not contacted, and the strategy was not peer reviewed by a second information specialist. Finally, the separation of contrast forms and all subgroup and robustness analyses were *post hoc* and should be regarded as exploratory.

### 4.6 Implications for research

The most valuable contribution future trials could make is size. Existing trials are individually too small to resolve moderate effects on biomarkers whose between-person variability is substantial; further small trials alone may not resolve the imprecision and between-trial uncertainty.

Beyond sample size, four reporting practices would materially improve the next synthesis. Trials should report both final values and change scores with complete dispersion data and the pre-post correlation, allowing one contrast form to be chosen consistently without reconstructing covariance. They should define control-group activity and background treatment precisely and apply any medication or care background equally across arms. They should register prospective, outcome-specific analysis plans (67), the absence of which raised concerns about selective reporting in almost every trial reviewed. And they should describe exercise prescriptions using a standard template with an explicit, comparable intensity metric (68), which would make dose-response analysis more feasible. Reporting numeric values rather than figures alone would allow every trial to contribute.

## 5 Conclusions

Very-low-certainty evidence means that the effects of resistance training and resistance-containing combined exercise on inflammatory biomarkers in adults living with HIV remain undetermined. Primary final-value meta-analyses for interleukin-6, tumor necrosis factor alpha, C-reactive protein, interleukin-1 beta and interleukin-10 all included no effect, and the interleukin-8 final-value evidence was limited to one inconclusive trial. Separate change-score analyses were also generally inconclusive; the one C-reactive protein change-score result that favored exercise came from a single trial and was not pooled. The CD4+ estimate of 124.2 cells/mm^3^ (54.6 to 193.7) suggests a possible increase, but it is not an established benefit: the evidence comes from seven small trials, none at low risk of bias, heterogeneity is substantial, and the prediction interval includes no effect. These findings do not weigh against exercise, which remains recommended on other well-established grounds; they show that its inflammatory and immunological consequences have not yet been established. Adequately powered trials with well-defined non-exercise comparators, balanced background treatment, prospectively registered analysis plans, standardized intensity reporting, and complete contrast-form data are required.

## Data Availability

The original contributions presented in the study are included in the article/**Supplementary Material**. Further inquiries can be directed to the corresponding authors.

## References

[1] Deeks SG . HIV infection, inflammation, immunosenescence, and aging. Annu Rev Med. (2011) 62:141–55. doi: 10.1146/annurev-med-042909-093756 21090961 PMC3759035

[2] Deeks SG Lewin SR Havlir DV . The end of AIDS: HIV infection as a chronic disease. Lancet. (2013) 382:1525–33. doi: 10.1016/s0140-6736(13)61809-7 24152939 PMC4058441

[3] Zicari S Sessa L Cotugno N Ruggiero A Morrocchi E Concato C . Immune activation, inflammation, and non-AIDS co-morbidities in HIV-infected patients under long-term ART. Viruses. (2019) 11:200. doi: 10.3390/v11030200 30818749 PMC6466530

[4] Kuller LH Tracy R Belloso W De Wit S Drummond F Lane HC . Inflammatory and coagulation biomarkers and mortality in patients with HIV infection. PLoS Med. (2008) 5:e203. doi: 10.1371/journal.pmed.0050203 18942885 PMC2570418

[5] Tenorio AR Zheng Y Bosch RJ Krishnan S Rodriguez B Hunt PW . Soluble markers of inflammation and coagulation but not T-cell activation predict non-AIDS-defining morbid events during suppressive antiretroviral treatment. J Infect Dis. (2014) 210:1248–59. doi: 10.1093/infdis/jiu254 24795473 PMC4192039

[6] Hunt PW Lee SA Siedner MJ . Immunologic biomarkers, morbidity, and mortality in treated HIV infection. J Infect Dis. (2016) 214:S44–50. doi: 10.1093/infdis/jiw275 27625430 PMC5021241

[7] Nou E Lo J Grinspoon SK . Inflammation, immune activation, and cardiovascular disease in HIV. AIDS. (2016) 30:1495–509. doi: 10.1097/qad.0000000000001109 27058351 PMC4889507

[8] Serrano-Villar S Sainz T Lee SA Hunt PW Sinclair E Shacklett BL . HIV-infected individuals with low CD4/CD8 ratio despite effective antiretroviral therapy exhibit altered T cell subsets, heightened CD8+ T cell activation, and increased risk of non-AIDS morbidity and mortality. PLoS Pathog. (2014) 10:e1004078. doi: 10.1371/journal.ppat.1004078 24831517 PMC4022662

[9] Gleeson M Bishop NC Stensel DJ Lindley MR Mastana SS Nimmo MA . The anti-inflammatory effects of exercise: mechanisms and implications for the prevention and treatment of disease. Nat Rev Immunol. (2011) 11:607–15. doi: 10.1038/nri3041 21818123

[10] Pedersen BK Saltin B . Exercise as medicine - evidence for prescribing exercise as therapy in 26 different chronic diseases. Scand J Med Sci Sports. (2015) 25:1–72. doi: 10.1111/sms.12581 26606383

[11] Pedersen BK Febbraio MA . Muscles, exercise and obesity: skeletal muscle as a secretory organ. Nat Rev Endocrinol. (2012) 8:457–65. doi: 10.1038/nrendo.2012.49 22473333

[12] O'Brien KK Tynan AM Nixon SA Glazier RH . Effectiveness of aerobic exercise for adults living with HIV: systematic review and meta-analysis using the Cochrane Collaboration protocol. BMC Infect Dis. (2016) 16:182. doi: 10.1186/s12879-016-1478-2 PMC4845358 27112335

[13] O'Brien KK Tynan AM Nixon SA Glazier RH . Effectiveness of progressive resistive exercise (PRE) in the context of HIV: systematic review and meta-analysis using the Cochrane Collaboration protocol. BMC Infect Dis. (2017) 17:268. doi: 10.1186/s12879-017-2342-8 PMC5389006 28403830

[14] Gomes-Neto M Conceicao CS Oliveira Carvalho V Brites C . A systematic review of the effects of different types of therapeutic exercise on physiologic and functional measurements in patients with HIV/AIDS. Clinics (Sao Paulo). (2013) 68:1157–67. doi: 10.6061/clinics/2013(08)16 24037014 PMC3752639

[15] Jaggers JR Hand GA . Health benefits of exercise for people living with HIV: a review of the literature. Am J Lifestyle Med. (2016) 10:184–92. doi: 10.1177/1559827614538750 PMC6124952 30202273

[16] Ibeneme SC Omeje C Myezwa H Ezeofor SN Anieto EM Irem F . Effects of physical exercises on inflammatory biomarkers and cardiopulmonary function in patients living with HIV: a systematic review with meta-analysis. BMC Infect Dis. (2019) 19:359. doi: 10.1186/s12879-019-3960-0 31035959 PMC6489236

[17] Poton R Polito M Farinatti P . Effects of resistance training in HIV-infected patients: a meta-analysis of randomised controlled trials. J Sports Sci. (2017) 35:2380–9. doi: 10.1080/02640414.2016.1267389 28001474

[18] Vingren JL Curtis JH Levitt DE Duplanty AA Lee EC McFarlin BK . Adding resistance training to the standard of care for inpatient substance abuse treatment in men with human immunodeficiency virus improves skeletal muscle health without altering cytokine concentrations. J Strength Cond Res. (2018) 32:76–82. doi: 10.1519/jsc.0000000000002289 29257793

[19] Vancampfort D Mugisha J De Hert M Probst M Firth J Gorczynski P . Global physical activity levels among people living with HIV: a systematic review and meta-analysis. Disabil Rehabil. (2018) 40:388–97. doi: 10.1080/09638288.2016.1260645 27929355

[20] Page MJ McKenzie JE Bossuyt PM Boutron I Hoffmann TC Mulrow CD . The PRISMA 2020 statement: an updated guideline for reporting systematic reviews. BMJ. (2021) 372:n71. doi: 10.1136/bmj.n71 33782057 PMC8005924

[21] Page MJ Moher D Bossuyt PM Boutron I Hoffmann TC Mulrow CD . PRISMA 2020 explanation and elaboration: updated guidance and exemplars for reporting systematic reviews. BMJ. (2021) 372:n160. doi: 10.1136/bmj.n160 33781993 PMC8005925

[22] Rethlefsen ML Kirtley S Waffenschmidt S Ayala AP Moher D Page MJ . PRISMA-S: an extension to the PRISMA statement for reporting literature searches in systematic reviews. Syst Rev. (2021) 10:39. doi: 10.1186/s13643-020-01542-z 33499930 PMC7839230

[23] Higgins JPT Thomas J Chandler J Cumpston M Li T Page MJ . Cochrane handbook for systematic reviews of interventions. Version 6.5. Higgins JPT Thomas J Chandler J Cumpston M Li T Page MJ , editors. London: Cochrane (2024).

[24] Cumpston M Li T Page MJ Chandler J Welch VA Higgins JP . Updated guidance for trusted systematic reviews: a new edition of the Cochrane handbook for systematic reviews of interventions. Cochrane Database Syst Rev. (2019) 10:ED000142. doi: 10.1002/14651858.ed000142 31643080 PMC10284251

[25] Hedges LV . Distribution theory for Glass's estimator of effect size and related estimators. J Educ Stat. (1981) 6:107–28. doi: 10.2307/1164588

[26] Viechtbauer W . Bias and efficiency of meta-analytic variance estimators in the random-effects model. J Educ Behav Stat. (2005) 30:261–93. doi: 10.3102/1076998603000326138293548

[27] IntHout J Ioannidis JP Borm GF . The Hartung-Knapp-Sidik-Jonkman method for random effects meta-analysis is straightforward and considerably outperforms the standard DerSimonian-Laird method. BMC Med Res Methodol. (2014) 14:25. doi: 10.1186/1471-2288-14-25 24548571 PMC4015721

[28] Higgins JP Thompson SG Deeks JJ Altman DG . Measuring inconsistency in meta-analyses. BMJ. (2003) 327:557–60. doi: 10.1136/bmj.327.7414.557 12958120 PMC192859

[29] IntHout J Ioannidis JP Rovers MM Goeman JJ . Plea for routinely presenting prediction intervals in meta-analysis. BMJ Open. (2016) 6:e010247. doi: 10.1136/bmjopen-2015-010247 27406637 PMC4947751

[30] Harris CR Millman KJ van der Walt SJ Gommers R Virtanen P Cournapeau D . Array programming with NumPy. Nature. (2020) 585:357–62. doi: 10.1038/s41586-020-2649-2 32939066 PMC7759461

[31] Virtanen P Gommers R Oliphant TE Haberland M Reddy T Cournapeau D . SciPy 1.0: fundamental algorithms for scientific computing in Python. Nat Methods. (2020) 17:261–72. doi: 10.1038/s41592-019-0686-2 32015543 PMC7056644

[32] Sterne JA Sutton AJ Ioannidis JP Terrin N Jones DR Lau J . Recommendations for examining and interpreting funnel plot asymmetry in meta-analyses of randomised controlled trials. BMJ. (2011) 343:d4002. doi: 10.1136/bmj.d4002 21784880

[33] Sterne JAC Savovic J Page MJ Elbers RG Blencowe NS Boutron I . RoB 2: a revised tool for assessing risk of bias in randomised trials. BMJ. (2019) 366:l4898. doi: 10.1136/bmj.l4898 31462531

[34] Sterne JA Hernan MA Reeves BC Savovic J Berkman ND Viswanathan M . ROBINS-I: a tool for assessing risk of bias in non-randomised studies of interventions. BMJ. (2016) 355:i4919. doi: 10.1136/bmj.i4919 27733354 PMC5062054

[35] Guyatt G Oxman AD Akl EA Kunz R Vist G Brozek J . GRADE guidelines: 1. Introduction-GRADE evidence profiles and summary of findings tables. J Clin Epidemiol. (2011) 64:383–94. doi: 10.1016/j.jclinepi.2010.04.026 21195583

[36] Balshem H Helfand M Schunemann HJ Oxman AD Kunz R Brozek J . GRADE guidelines: 3. Rating the quality of evidence. J Clin Epidemiol. (2011) 64:401–6. doi: 10.1016/j.jclinepi.2010.07.015 21208779

[37] Guyatt GH Oxman AD Kunz R Woodcock J Brozek J Helfand M . GRADE guidelines: 7. Rating the quality of evidence-inconsistency. J Clin Epidemiol. (2011) 64:1294–302. doi: 10.1016/j.jclinepi.2011.03.017 21803546

[38] Guyatt GH Oxman AD Kunz R Brozek J Alonso-Coello P Rind D . GRADE guidelines 6. Rating the quality of evidence-imprecision. J Clin Epidemiol. (2011) 64:1283–93. doi: 10.1016/j.jclinepi.2011.01.012 21839614

[39] Brito-Neto JG de Andrade MF de Almeida VD Paiva DCC de Morais NM Bezerra CM . Strength training improves body composition, muscle strength and increases CD4+ T lymphocyte levels in people living with HIV/AIDS. Infect Dis Rep. (2019) 11:7925. doi: 10.4081/idr.2019.7925 31205641 PMC6547026

[40] Dianatinasab M Fararouei M Padehban V Dianatinasab A Alimohamadi Y Beheshti S . The effect of a 12-week combinational exercise program on CD4 count and mental health among HIV infected women: a randomized control trial. J Exerc Sci Fit. (2018) 16:21–5. doi: 10.1016/j.jesf.2018.02.001 30662488 PMC6323156

[41] Dudgeon WD Jaggers JR Phillips KD Durstine JL Burgess SE Lyerly GW . Moderate-intensity exercise improves body composition and improves physiological markers of stress in HIV-infected men. ISRN AIDS. (2012) 2012:145127. doi: 10.5402/2012/145127 24052871 PMC3767244

[42] Ghayomzadeh M SeyedAlinaghi S Shamsi MM Rezaei S Earnest CP Akbarnejad S . Effect of 8 weeks of hospital-based resistance training program on TCD4+ cell count and anthropometric characteristics of patients with HIV. J Strength Cond Res. (2019) 33:1146–55. doi: 10.1519/jsc.0000000000002394 29219895

[43] Ghayomzadeh M Hackett D SeyedAlinaghi S Gholami M Hosseini Rouzbahani N Voltarelli FA . Combined training improves the diagnostic measures of sarcopenia and decreases the inflammation in HIV-infected individuals. J Cachexia Sarcopenia Muscle. (2022) 13:1024–35. doi: 10.1002/jcsm.12926 35142082 PMC8977956

[44] Ghayomzadeh M Earnest CP Hackett D SeyedAlinaghi SA Navalta JW Gholami M . Combination of resistance and aerobic exercise for six months improves bone mass and physical function in HIV-infected individuals: a randomized controlled trial. Scand J Med Sci Sports. (2021) 31:720–32. doi: 10.1111/sms.13871 33185897

[45] Gomes RD Borges JP Lima DB Farinatti PTV . Effects of physical exercise in the perception of life satisfaction and immunological function in HIV-infected patients: non-randomized clinical trial. Rev Bras Fisioter. (2010) 14:390–5. 21180864

[46] Khoramipour K Katanchi A Hajirasouli M Behbudi L Basereh A Dehghan P . Combined training in patient with AIDS: improved quality of life and preserved BDNF. Sport Sci Health. (2022) 18:39–46. doi: 10.1007/s11332-021-00771-6

[47] Pedro RE Candido N Guariglia DA Melo BP Bertolini DA Peres SB . Exercise improves cytokine profile in HIV-infected people. Cytokine. (2017) 9:18–23. doi: 10.1016/j.cyto.2017.06.019 28683357

[48] Guariglia DA Pedro RE Deminice R Rosa FT Peres SB Franzoi De Moraes SM . Effect of combined training on body composition and metabolic variables in people living with HIV. Cytokine. (2018) 111:505–10. doi: 10.1016/j.cyto.2018.05.028 29903591

[49] Piraux E Reychler G Forget P Yombi JC Caty G . Feasibility and preliminary effects of a telerehabilitation program for people living with HIV: a pilot randomized study. J Assoc Nurses AIDS Care. (2019) 30:176–85. doi: 10.1097/jnc.0000000000000005 30822290

[50] Ryan AS Briggs BC Lozano AJ Iranmanesh A Oursler KK . Randomized clinical trial of high intensity exercise in people with HIV: effects on muscle composition and inflammation. J Acquir Immune Defic Syndr. (2025) 99:492–501. doi: 10.1097/qai.0000000000003692 40338873 PMC13267803

[51] Soares VL Soares WF Zanetti HR Neves FF Silva-Vergara ML Mendes EL . Daily undulating periodization is more effective than nonperiodized training on maximal strength, aerobic capacity, and TCD4+ cell count in people living with HIV. J Strength Cond Res. (2022) 36:1738–48. doi: 10.1519/JSC.0000000000003675 32604148

[52] Yarasheski KE Cade WT Overton ET Mondy KE Hubert S Laciny E . Exercise training augments the peripheral insulin-sensitizing effects of pioglitazone in HIV-infected adults with insulin resistance and central adiposity. Am J Physiol Endocrinol Metab. (2011) 300:E243–51. doi: 10.1152/ajpendo.00468.2010 20959530 PMC3023206

[53] Zanetti HR da Cruz LG Lourenco CLM Neves FF Silva-Vergara ML Mendes EL . Non-linear resistance training reduces inflammatory biomarkers in persons living with HIV: a randomized controlled trial. Eur J Sport Sci. (2016) 16:1232–9. doi: 10.1080/17461391.2016.1167962 27027897

[54] Zanetti HR Cruz LG Lourenco CL Ribeiro GC Ferreira de Jesus Leite MA Neves FF . Nonlinear resistance training enhances the lipid profile and reduces inflammation marker in people living with HIV: a randomized clinical trial. J Phys Act Health. (2016) 13:765–70. doi: 10.1123/jpah.2015-0540 26901242

[55] Zanetti HR Goncalves A Teixeira Paranhos Lopes L Mendes EL Roever L Silva-Vergara ML . Effects of exercise training and statin use in people living with human immunodeficiency virus with dyslipidemia. Med Sci Sports Exerc. (2020) 52:16–24. doi: 10.1249/MSS.0000000000002120 31834252

[56] Petersen AM Pedersen BK . The anti-inflammatory effect of exercise. J Appl Physiol. (2005) 98:1154–62. doi: 10.1152/japplphysiol.00164.2004 15772055

[57] Fischer CP . Interleukin-6 in acute exercise and training: what is the biological relevance? Exerc Immunol Rev. (2006) 12:6–33. 17201070

[58] Nash MS . Exercise and immunology. Med Sci Sports Exerc. (1994) 26:125–7. doi: 10.1249/00005768-199402000-00001 8164528

[59] Simpson RJ Kunz H Agha N Graff R . Exercise and the regulation of immune functions. Prog Mol Biol Transl Sci. (2015) 135:355–80. doi: 10.1016/bs.pmbts.2015.08.001 26477922

[60] Campbell JP Turner JE . Debunking the myth of exercise-induced immune suppression: redefining the impact of exercise on immunological health across the lifespan. Front Immunol. (2018) 9:648. doi: 10.3389/fimmu.2018.00648 29713319 PMC5911985

[61] Turner RM Bird SM Higgins JP . The impact of study size on meta-analyses: examination of underpowered studies in Cochrane reviews. PLoS One. (2013) 8:e59202. doi: 10.1371/journal.pone.0059202 23544056 PMC3609745

[62] Button KS Ioannidis JP Mokrysz C Nosek BA Flint J Robinson ES . Power failure: why small sample size undermines the reliability of neuroscience. Nat Rev Neurosci. (2013) 14:365–76. doi: 10.1038/nrn3475 23571845

[63] Nakagawa S Cuthill IC . Effect size, confidence interval and statistical significance: a practical guide for biologists. Biol Rev Camb Philos Soc. (2007) 82:591–605. doi: 10.1111/j.1469-185x.2007.00027.x 17944619

[64] Bull FC Al-Ansari SS Biddle S Borodulin K Buman MP Cardon G . World Health Organization 2020 guidelines on physical activity and sedentary behaviour. Br J Sports Med. (2020) 54:1451–62. doi: 10.1136/bjsports-2020-102955 33239350 PMC7719906

[65] Ridker PM Rifai N Rose L Buring JE Cook NR . Comparison of C-reactive protein and low-density lipoprotein cholesterol levels in the prediction of first cardiovascular events. N Engl J Med. (2002) 347:1557–65. doi: 10.1056/nejmoa021993 12432042

[66] Ridker PM Everett BM Thuren T MacFadyen JG Chang WH Ballantyne C . Antiinflammatory therapy with canakinumab for atherosclerotic disease. N Engl J Med. (2017) 377:1119–31. doi: 10.1056/nejmoa1707914 28845751

[67] Chan AW Tetzlaff JM Altman DG Laupacis A Gotzsche PC Krleza-Jeric K . SPIRIT 2013 statement: defining standard protocol items for clinical trials. Ann Intern Med. (2013) 158:200–7. doi: 10.7326/0003-4819-158-3-201302050-00583 23295957 PMC5114123

[68] Slade SC Dionne CE Underwood M Buchbinder R . Consensus on Exercise Reporting Template (CERT): explanation and elaboration statement. Br J Sports Med. (2016) 50:1428–37. doi: 10.1136/bjsports-2016-096651 27707738

