# Supplementary material for: Resistance-containing exercise versus non-exercise control for inflammatory markers and T-cell counts in adults living with HIV: a systematic review and meta-analysis
Source: Front Immunol. 2026 Oct 7;17:1949799. doi: 10.3389/fimmu.2026.1949799 (PMC13683185; doi:10.3389/fimmu.2026.1949799)
Supplement: Supplementary file 1 [file DataSheet1.docx]

Supplementary Material (v5)

**Contents**

Table S1. Full electronic search strategies.

Table S2. Outcome-level data used to compute every effect estimate.

Table S3. Domain-level risk-of-bias judgments with supporting reasoning.

Table S4. Sensitivity analyses with Primary and Supporting reference estimates.

Table S5. Post hoc subgroup analyses, separate contrast-form analyses, and screening of candidate moderators.

Table S6. GRADE certainty of evidence, by construct and domain.

Table S7. Constructs reported but not pooled, and the reason for narrative synthesis.

Figure S1. Primary final-value forest plot of Interleukin-6 (IL-6).

Figure S2. Primary final-value forest plot of Tumor necrosis factor alpha (TNF-α).

Figure S3. Primary final-value forest plot of C-reactive protein (CRP).

Figure S4. Primary final-value forest plot of Interleukin-1 beta (IL-1β).

Figure S5. Individual final-value result for Interleukin-8 (IL-8).

Figure S6. Primary final-value forest plot of Interleukin-10 (IL-10).

Figure S7. Subgroup analyses for CD4+ T-cell count.

Figure S8. Post hoc robustness analyses for the revised primary and supporting estimates.

Figure S9. Availability of outcome data across trials and constructs.

**Table S1. Full electronic search strategies.**

| **Database** | **Database-specific search strategy** | **Date searched** | **Records retrieved** |
| --- | --- | --- | --- |
| #1 PubMed | ("HIV"[MeSH Terms] OR "HIV Infections"[MeSH Terms] OR "Acquired Immunodeficiency Syndrome"[MeSH Terms] OR HIV[tiab] OR "human immunodeficiency virus"[tiab] OR AIDS[tiab] OR "people living with HIV"[tiab] OR PLWH[tiab] OR PLHIV[tiab] OR seropositive*[tiab])  AND ("Resistance Training"[MeSH Terms] OR "Exercise"[MeSH Terms] OR "Exercise Therapy"[MeSH Terms] OR "Physical Conditioning, Human"[MeSH Terms] OR "resistance training"[tiab] OR "strength training"[tiab] OR "weight training"[tiab] OR "weight lifting"[tiab] OR "weight-bearing exercise"[tiab] OR "progressive resistance"[tiab] OR "elastic band*"[tiab] OR "concurrent training"[tiab] OR "combined training"[tiab] OR "combined exercise"[tiab] OR "multimodal exercise"[tiab] OR "aerobic and resistance"[tiab] OR "exercise training"[tiab] OR "physical training"[tiab])  AND ("Inflammation"[MeSH Terms] OR "Cytokines"[MeSH Terms] OR "Interleukins"[MeSH Terms] OR "C-Reactive Protein"[MeSH Terms] OR "Tumor Necrosis Factor-alpha"[MeSH Terms] OR "CD4 Lymphocyte Count"[MeSH Terms] OR "CD4-Positive T-Lymphocytes"[MeSH Terms] OR "CD8-Positive T-Lymphocytes"[MeSH Terms] OR "Viral Load"[MeSH Terms] OR inflammat*[tiab] OR cytokine*[tiab] OR interleukin*[tiab] OR "IL-6"[tiab] OR "IL-8"[tiab] OR "IL-10"[tiab] OR "IL-1beta"[tiab] OR "C-reactive protein"[tiab] OR CRP[tiab] OR "tumor necrosis factor"[tiab] OR "tumor necrosis factor"[tiab] OR "TNF-alpha"[tiab] OR "CD4"[tiab] OR "CD8"[tiab] OR "T-cell count*"[tiab] OR "viral load"[tiab] OR immun*[tiab])  AND ("Randomized Controlled Trial"[Publication Type] OR "Controlled Clinical Trial"[Publication Type] OR "Random Allocation"[MeSH Terms] OR randomi*[tiab] OR "controlled trial"[tiab] OR "clinical trial"[tiab] OR "control group"[tiab] OR "usual care"[tiab] OR "wait list"[tiab] OR "waiting list"[tiab])  Filters: publication date to 1 June 2026; no language restriction. | 1 June 2026 | 138 |
| #2 Web of Science Core Collection | TS=(HIV OR "human immunodeficiency virus" OR AIDS OR "people living with HIV" OR PLWH OR PLHIV OR seropositive*)  AND TS=("resistance training" OR "strength training" OR "weight training" OR "weight lifting" OR "progressive resistance" OR "elastic band*" OR "concurrent training" OR "combined training" OR "combined exercise" OR "multimodal exercise" OR "aerobic and resistance" OR "exercise training" OR "physical training" OR "exercise therapy")  AND TS=(inflammat* OR cytokine* OR interleukin* OR "IL-6" OR "IL-8" OR "IL-10" OR "IL-1beta" OR "C-reactive protein" OR CRP OR "tumor necrosis factor" OR "tumor necrosis factor" OR "TNF-alpha" OR CD4 OR CD8 OR "T-cell count*" OR "viral load" OR immun*)  AND TS=(randomi* OR "controlled trial" OR "clinical trial" OR "control group" OR "usual care" OR "wait list" OR "waiting list")  Timespan: all years to 1 June 2026; document types not restricted. | 1 June 2026 | 116 |
| #3 Embase (Elsevier, embase.com) | ('human immunodeficiency virus infection'/exp OR 'human immunodeficiency virus'/exp OR (HIV OR 'human immunodeficiency virus' OR AIDS OR PLWH OR PLHIV OR seropositive*):ti,ab,kw) AND ('resistance training'/exp OR 'exercise'/exp OR 'kinesiotherapy'/exp OR ('resistance training' OR 'strength training' OR 'weight training' OR 'weight lifting' OR 'progressive resistance' OR 'elastic band*' OR 'concurrent training' OR 'combined training' OR 'combined exercise' OR 'multimodal exercise' OR 'aerobic and resistance' OR 'exercise training' OR 'physical training'):ti,ab,kw) AND ('inflammation'/exp OR 'cytokine'/exp OR 'interleukin'/exp OR 'C reactive protein'/exp OR 'tumor necrosis factor alpha'/exp OR 'CD4 lymphocyte count'/exp OR 'virus load'/exp OR (inflammat* OR cytokine* OR interleukin* OR 'IL-6' OR 'IL-8' OR 'IL-10' OR 'IL-1beta' OR 'C-reactive protein' OR CRP OR 'tumor necrosis factor' OR 'TNF-alpha' OR CD4 OR CD8 OR 'T-cell count*' OR 'viral load' OR immun*):ti,ab,kw) AND ('randomized controlled trial'/exp OR 'controlled clinical trial'/exp OR (randomi* OR 'controlled trial' OR 'clinical trial' OR 'control group' OR 'usual care' OR 'wait list' OR 'waiting list'):ti,ab,kw)  Limiters: records added to 1 June 2026; no language limiter applied. | 1 June 2026 | 457 |
| #4 Cochrane Library (CENTRAL) | (HIV OR "human immunodeficiency virus" OR AIDS OR PLWH OR PLHIV OR seropositive*):ti,ab,kw AND ("resistance training" OR "strength training" OR "weight training" OR "weight lifting" OR "progressive resistance" OR (elastic NEXT band*) OR "concurrent training" OR "combined training" OR "combined exercise" OR "aerobic and resistance" OR "exercise training" OR "physical training"):ti,ab,kw AND (inflammat* OR cytokine* OR interleukin* OR "C reactive protein" OR CRP OR "tumor necrosis factor" OR "TNF alpha" OR CD4 OR CD8 OR (T NEXT cell* NEXT count*) OR "viral load" OR immun*):ti,ab,kw  Limiters: published before 1 June 2026; no language limiter applied. | 1 June 2026 | 143 |
| #5 Scopus | TITLE-ABS-KEY(HIV OR "human immunodeficiency virus" OR AIDS OR PLWH OR PLHIV OR seropositive*)  AND TITLE-ABS-KEY("resistance training" OR "strength training" OR "weight training" OR "weight lifting" OR "progressive resistance" OR "elastic band*" OR "concurrent training" OR "combined training" OR "combined exercise" OR "multimodal exercise" OR "aerobic and resistance" OR "exercise training" OR "physical training")  AND TITLE-ABS-KEY(inflammat* OR cytokine* OR interleukin* OR "C-reactive protein" OR CRP OR "tumor necrosis factor" OR "tumor necrosis factor" OR "TNF-alpha" OR CD4 OR CD8 OR "T-cell count*" OR "viral load" OR immun*)  AND TITLE-ABS-KEY(randomi* OR "controlled trial" OR "clinical trial" OR "control group" OR "usual care" OR "wait list" OR "waiting list")  AND PUBDATETXT BEFORE "June 2026" | 1 June 2026 | 155 |
| #6 SPORTDiscus (EBSCOhost) | (TI(HIV OR "human immunodeficiency virus" OR "HIV infection*" OR "HIV-infected" OR "HIV positive" OR "HIV-positive" OR AIDS OR PLWH OR PLHIV OR PLWHA OR "people living with HIV" OR "persons living with HIV" OR "acquired immunodeficiency syndrome") OR AB(HIV OR "human immunodeficiency virus" OR "HIV infection*" OR "HIV-infected" OR "HIV positive" OR "HIV-positive" OR AIDS OR PLWH OR PLHIV OR PLWHA OR "people living with HIV" OR "persons living with HIV" OR "acquired immunodeficiency syndrome") OR SU(HIV OR "human immunodeficiency virus" OR AIDS)) AND (TI("resistance training" OR "resistance exercise*" OR "progressive resistance" OR "progressive resistive" OR "strength training" OR "strength exercise*" OR "strengthening exercise*" OR "muscle strengthening" OR "weight training" OR "weight lifting" OR weightlifting OR "elastic band*" OR "concurrent training" OR "concurrent exercise*" OR "combined training" OR "combined exercise*" OR "multimodal exercise*" OR "combinational exercise*" OR (aerobic N5 resistance) OR (aerobic N5 strength) OR (endurance N5 resistance) OR (endurance N5 strength)) OR AB("resistance training" OR "resistance exercise*" OR "progressive resistance" OR "progressive resistive" OR "strength training" OR "strength exercise*" OR "strengthening exercise*" OR "muscle strengthening" OR "weight training" OR "weight lifting" OR weightlifting OR "elastic band*" OR "concurrent training" OR "concurrent exercise*" OR "combined training" OR "combined exercise*" OR "multimodal exercise*" OR "combinational exercise*" OR (aerobic N5 resistance) OR (aerobic N5 strength) OR (endurance N5 resistance) OR (endurance N5 strength))) AND (TI(random* OR trial* OR "controlled study" OR "controlled studies" OR "controlled trial*" OR "clinical trial*" OR "control group*" OR "comparison group*" OR "usual care" OR "non-exercise control" OR waitlist OR "wait list" OR "waiting list") OR AB(random* OR trial* OR "controlled study" OR "controlled studies" OR "controlled trial*" OR "clinical trial*" OR "control group*" OR "comparison group*" OR "usual care" OR "non-exercise control" OR waitlist OR "wait list" OR "waiting list"))  Limiters: published before 1 June 2026; no language limiter applied. | 1 June 2026 | 149 |

Searches were conducted in six databases on 1 June 2026. Table S1 presents the database-specific search strategies reconstructed from the contemporaneous search records and formatted in the syntax of the corresponding database, together with the recorded search date and number of records retrieved. The six database searches yielded 1,158 records in total. Concept blocks combined population, intervention, outcome and study-design terms, using controlled vocabulary where supported together with free-text terms. The Cochrane Library strategy used free-text terms only, and the SPORTDiscus strategy omitted a separate outcome block to reduce the risk of missing records because biochemical outcomes are inconsistently indexed in that database. No language restriction was applied.

Reference lists of the included reports and of relevant reviews were screened in addition to the database searches. Trial registries were not searched as a separate information source; registry records were covered in part through the Cochrane Library (CENTRAL), which incorporates records from ClinicalTrials.gov and the World Health Organization International Clinical Trials Registry Platform on a monthly basis. The reasons for this decision, and its consequences for the interpretation of the review, are set out in Sections 2.3 and 4.5 of the main text. Authors of included reports were not contacted, and the strategy was not peer reviewed by a second information specialist.

AB, abstract field; ti,ab,kw, title, abstract and keyword fields; /exp, Emtree term exploded; MeSH, Medical Subject Headings; N5 and NEXT, proximity operators; SU, subject-heading field; TI, title field; TITLE-ABS-KEY, title, abstract and keyword fields; TS, topic field.

**Table S2. Outcome-level data used to compute every effect estimate.**

| **Outcome** | **Trial** | **Form** | **n int/comp** | **Intervention, mean (SD)** | **Comparator, mean (SD)** | **Unit** | **Metric** | **Effect (95% CI)** | **SD source** | **Role** |
| --- | --- | --- | --- | --- | --- | --- | --- | --- | --- | --- |
| Interleukin-6 (IL-6) | Dudgeon et al. (2012) | Final value | 16/10 | 3.10 (2.40) | 6.70 (6.32) | pg/mL | SMD | −0.81 (−1.60 to −0.01) | SE | Primary |
| Interleukin-6 (IL-6) | Ghayomzadeh et al. (2022) | Change score | 20/20 | −1.30 (0.49) | 1.21 (0.99) | pg/mL | SMD | −3.15 (−4.07 to −2.23) | Reported | Supporting |
| Interleukin-6 (IL-6) | Pedro et al. (2017) | Final value | 17/11 | 2.10 (1.10) | 2.40 (1.00) | pg/mL | SMD | −0.27 (−1.01 to 0.47) | Reported | Primary |
| Interleukin-6 (IL-6) | Ryan et al. (2025) | Final value | 15/13 | 2.50 (1.43) | 2.90 (2.20) | pg/mL | SMD | −0.21 (−0.94 to 0.51) | IQR | Primary |
| Interleukin-6 (IL-6) | Vingren et al. (2018) | Final value | 8/8 | 3.90 (1.80) | 2.70 (1.30) | pg/mL | SMD | 0.72 (−0.24 to 1.68) | Reported | Primary |
| Interleukin-6 (IL-6) | Zanetti et al. (2016) | Change score | 15/15 | −2.30 (0.40) | 0.09 (0.30) | pg/mL | SMD | −6.58 (−8.38 to −4.77) | Reported | Supporting |
| Interleukin-6 (IL-6) | Zanetti et al. (2020) | Change score | 18/18 | −0.92 (1.00) | 0.03 (2.50) | pg/dL | SMD | −0.49 (−1.14 to 0.16) | Reported | Supporting |
| Interleukin-6 (IL-6) | Zanetti et al. (2020) | Change score | 18/18 | −4.47 (2.42) | −0.74 (2.20) | pg/dL | SMD | −1.58 (−2.31 to −0.84) | Reported | Sensitivity |
| Interleukin-6 (IL-6) | Zanetti et al. (2020) | Change score | 36/36 | −2.69 (2.56) | −0.35 (2.35) | pg/dL | SMD | −0.94 (−1.42 to −0.46) | Reported | Sensitivity |
| Tumor necrosis factor alpha (TNF-α) | Ghayomzadeh et al. (2022) | Change score | 20/20 | −1.41 (0.71) | 1.87 (1.40) | pg/mL | SMD | −2.90 (−3.77 to −2.02) | Reported | Supporting |
| Tumor necrosis factor alpha (TNF-α) | Pedro et al. (2017) | Final value | 17/11 | 2.60 (1.80) | 3.60 (3.10) | pg/mL | SMD | −0.41 (−1.15 to 0.34) | Reported | Primary |
| Tumor necrosis factor alpha (TNF-α) | Ryan et al. (2025) | Final value | 15/13 | 4.15 (2.90) | 4.23 (1.21) | pg/mL | SMD | −0.03 (−0.76 to 0.69) | IQR | Primary |
| Tumor necrosis factor alpha (TNF-α) | Vingren et al. (2018) | Final value | 8/8 | 15.40 (8.00) | 9.90 (3.70) | pg/mL | SMD | 0.83 (−0.14 to 1.80) | Reported | Primary |
| Tumor necrosis factor alpha (TNF-α) | Zanetti et al. (2016) | Change score | 15/15 | −5.72 (2.70) | 1.33 (1.30) | pg/mL | SMD | −3.24 (−4.31 to −2.16) | Reported | Supporting |
| C-reactive protein (CRP) | Pedro et al. (2017) | Final value | 12/9 | 488.00 (559.70) | 367.30 (292.40) | pg/mL | SMD | 0.25 (−0.58 to 1.08) | Reported | Primary |
| C-reactive protein (CRP) | Piraux et al. (2019) | Final value | 9/8 | 3.00 (2.62) | 2.00 (1.79) | mg/L | SMD | 0.42 (−0.50 to 1.33) | IQR | Primary |
| C-reactive protein (CRP) | Ryan et al. (2025) | Final value | 15/13 | 2.09 (1.33) | 5.63 (8.11) | mg/L | SMD | −0.61 (−1.35 to 0.13) | IQR | Primary |
| C-reactive protein (CRP) | Zanetti et al. (2020) | Change score | 18/18 | −1.44 (1.10) | 0.01 (0.50) | mg/dL | SMD | −1.66 (−2.40 to −0.91) | Reported | Supporting |
| C-reactive protein (CRP) | Zanetti et al. (2020) | Change score | 18/18 | −1.47 (1.00) | −1.44 (1.60) | mg/dL | SMD | −0.02 (−0.66 to 0.62) | Reported | Sensitivity |
| C-reactive protein (CRP) | Zanetti et al. (2020) | Change score | 36/36 | −1.45 (1.04) | −0.71 (1.38) | mg/dL | SMD | −0.60 (−1.07 to −0.13) | Reported | Sensitivity |
| Interleukin-1 beta (IL-1β) | Dudgeon et al. (2012) | Final value | 16/10 | 7.50 (6.80) | 6.10 (1.58) | pg/mL | SMD | 0.25 (−0.52 to 1.02) | SE | Primary |
| Interleukin-1 beta (IL-1β) | Vingren et al. (2018) | Final value | 8/8 | 0.10 (0.10) | 0.40 (0.30) | pg/mL | SMD | −1.27 (−2.29 to −0.24) | Reported | Primary |
| Interleukin-1 beta (IL-1β) | Zanetti et al. (2016) | Change score | 15/15 | −1.10 (0.60) | 0.19 (0.44) | pg/mL | SMD | −2.39 (−3.31 to −1.46) | Reported | Supporting |
| Interleukin-1 beta (IL-1β) | Zanetti et al. (2020) | Change score | 18/18 | −4.27 (2.80) | 0.02 (1.90) | pg/dL | SMD | −1.75 (−2.51 to −1.00) | Reported | Supporting |
| Interleukin-1 beta (IL-1β) | Zanetti et al. (2020) | Change score | 18/18 | −7.95 (2.60) | −4.02 (3.40) | pg/dL | SMD | −1.27 (−1.97 to −0.57) | Reported | Sensitivity |
| Interleukin-1 beta (IL-1β) | Zanetti et al. (2020) | Change score | 36/36 | −6.11 (3.25) | −2.00 (3.40) | pg/dL | SMD | −1.22 (−1.72 to −0.72) | Reported | Sensitivity |
| Interleukin-8 (IL-8) | Pedro et al. (2017) | Final value | 17/11 | 5.40 (2.30) | 8.10 (6.00) | pg/mL | SMD | −0.63 (−1.39 to 0.12) | Reported | Primary |
| Interleukin-8 (IL-8) | Zanetti et al. (2016) | Change score | 15/15 | −5.50 (3.40) | −0.15 (2.30) | pg/mL | SMD | −1.79 (−2.62 to −0.96) | Reported | Supporting |
| Interleukin-8 (IL-8) | Zanetti et al. (2020) | Change score | 18/18 | −0.36 (0.30) | −0.04 (0.70) | pg/dL | SMD | −0.58 (−1.23 to 0.07) | Reported | Supporting |
| Interleukin-8 (IL-8) | Zanetti et al. (2020) | Change score | 18/18 | −0.71 (1.46) | −0.39 (0.50) | pg/dL | SMD | −0.29 (−0.93 to 0.36) | Reported | Sensitivity |
| Interleukin-8 (IL-8) | Zanetti et al. (2020) | Change score | 36/36 | −0.53 (1.05) | −0.21 (0.63) | pg/dL | SMD | −0.37 (−0.83 to 0.10) | Reported | Sensitivity |
| Interleukin-10 (IL-10) | Pedro et al. (2017) | Final value | 17/11 | 7.10 (0.40) | 7.60 (0.80) | pg/mL | SMD | −0.83 (−1.59 to −0.06) | Reported | Primary |
| Interleukin-10 (IL-10) | Vingren et al. (2018) | Final value | 8/8 | 108.50 (67.00) | 58.20 (17.80) | pg/mL | SMD | 0.97 (−0.02 to 1.96) | Reported | Primary |
| Interleukin-10 (IL-10) | Zanetti et al. (2016) | Change score | 15/15 | 1.00 (0.40) | −0.05 (0.13) | pg/mL | SMD | 3.44 (2.32 to 4.55) | Reported | Supporting |
| Interleukin-10 (IL-10) | Zanetti et al. (2020) | Change score | 18/18 | 0.90 (0.70) | −0.03 (0.60) | pg/dL | SMD | 1.39 (0.68 to 2.11) | Reported | Supporting |
| Interleukin-10 (IL-10) | Zanetti et al. (2020) | Change score | 18/18 | 0.97 (0.61) | 0.41 (0.40) | pg/dL | SMD | 1.06 (0.38 to 1.75) | Reported | Sensitivity |
| Interleukin-10 (IL-10) | Zanetti et al. (2020) | Change score | 36/36 | 0.94 (0.65) | 0.19 (0.55) | pg/dL | SMD | 1.23 (0.73 to 1.73) | Reported | Sensitivity |
| CD4+ T-cell count | Brito-Neto et al. (2019) | Final value | 9/10 | 559.67 (61.98) | 449.30 (37.28) | cells/mm³ | MD | 110.4 (63.7 to 157.0) | Reported | Primary |
| CD4+ T-cell count | Dianatinasab et al. (2018) | Final value | 14/16 | 495.57 (149.37) | 491.18 (284.48) | cells/mm³ | MD | 4.4 (−155.5 to 164.2) | Reported | Primary |
| CD4+ T-cell count | Ghayomzadeh et al. (2019) | Final value | 12/6 | 578.17 (211.03) | 269.33 (148.79) | cells/mm³ | MD | 308.8 (140.2 to 477.5) | Reported | Primary |
| CD4+ T-cell count | Gomes et al. (2010) | Final value | 19/10 | 565.60 (72.10) | 398.10 (69.40) | cells/mm³ | MD | 167.5 (113.6 to 221.4) | Reported | Primary |
| CD4+ T-cell count | Piraux et al. (2019) | Final value | 9/8 | 746.40 (201.70) | 612.50 (200.30) | cells/mm³ | MD | 133.9 (−57.5 to 325.3) | Reported | Primary |
| CD4+ T-cell count | Soares et al. (2022) | Change score | 26/15 | 134.30 (163.01) | −18.20 (49.40) | cells/mm³ | MD | 152.5 (85.0 to 220.0) | Reported | Primary |
| CD4+ T-cell count | Zanetti et al. (2016) | Change score | 15/15 | 64.40 (38.50) | 6.20 (59.50) | cells/mm³ | MD | 58.2 (22.3 to 94.1) | Reported | Primary |
| Adiponectin | Ghayomzadeh et al. (2022) | Change score | 20/20 | 1.41 (0.82) | −1.39 (0.73) | µg/mL | SMD | 3.54 (2.55 to 4.52) | Reported | Narrative |
| Adiponectin | Yarasheski et al. (2011) | Final value | 19/20 | 6.50 (3.92) | 7.00 (3.13) | µg/mL | SMD | −0.14 (−0.75 to 0.48) | SE | Narrative |
| CD4+ T-cell percentage | Gomes et al. (2010) | Final value | 19/10 | 23.50 (2.00) | 21.70 (3.50) | % | MD | 1.8 (−0.5 to 4.1) | Reported | Narrative |
| CD8+ T-cell count | Soares et al. (2022) | Change score | 26/15 | 105.90 (269.13) | −36.50 (95.80) | cells/mm³ | MD | 142.4 (28.2 to 256.6) | Reported | Narrative |
| CD8+ T-cell count | Zanetti et al. (2016) | Change score | 15/15 | 186.60 (78.70) | 3.80 (36.10) | cells/mm³ | MD | 182.8 (139.0 to 226.6) | Reported | Narrative |
| Cortisol | Dudgeon et al. (2012) | Final value | 16/10 | 61.20 (36.00) | 57.70 (27.83) | µg/mL | SMD | 0.10 (−0.66 to 0.87) | SE | Narrative |
| Cortisol | Vingren et al. (2018) | Final value | 8/8 | 617.00 (132.00) | 720.00 (218.00) | nmol/L | SMD | −0.54 (−1.49 to 0.40) | Reported | Narrative |
| D-dimer | Piraux et al. (2019) | Final value | 9/8 | 300.93 (133.65) | 416.43 (239.64) | ng/mL | SMD | −0.58 (−1.50 to 0.35) | IQR | Narrative |
| Fibrinogen | Zanetti et al. (2020) | Change score | 18/18 | −52.60 (25.40) | 0.66 (10.40) | mg/dL | SMD | −2.68 (−3.57 to −1.79) | Reported | Narrative |
| Fibrinogen | Zanetti et al. (2020) | Change score | 18/18 | −64.70 (38.80) | −48.83 (31.40) | mg/dL | SMD | −0.44 (−1.09 to 0.21) | Reported | Narrative |
| Fibrinogen | Zanetti et al. (2020) | Change score | 36/36 | −58.65 (32.90) | −24.09 (34.08) | mg/dL | SMD | −1.02 (−1.51 to −0.53) | Reported | Narrative |
| Granulocyte-macrophage colony-stimulating factor (GM-CSF) | Pedro et al. (2017) | Final value | 17/11 | 4.10 (0.30) | 4.50 (0.90) | pg/mL | SMD | −0.64 (−1.40 to 0.11) | Reported | Narrative |
| Growth hormone (GH) | Dudgeon et al. (2012) | Final value | 16/10 | 7.10 (4.80) | 18.90 (37.31) | ng/day | SMD | −0.49 (−1.27 to 0.28) | SE | Narrative |
| Insulin-like growth factor 1 (IGF-1) | Dudgeon et al. (2012) | Final value | 16/10 | 132.00 (43.20) | 137.00 (55.34) | ng/mL | SMD | −0.10 (−0.87 to 0.66) | SE | Narrative |
| Insulin-like growth factor 1 (IGF-1) | Ghayomzadeh et al. (2022) | Change score | 20/20 | 74.36 (56.64) | 7.19 (99.00) | pg/mL | SMD | 0.82 (0.18 to 1.45) | Reported | Narrative |
| Insulin-like growth factor-binding protein 3 (IGFBP-3) | Dudgeon et al. (2012) | Final value | 16/10 | 1934.00 (3484.00) | 1769.00 (689.38) | ng/mL | SMD | 0.06 (−0.71 to 0.82) | SE | Narrative |
| Interferon gamma (IFN-γ) | Pedro et al. (2017) | Final value | 17/11 | 2.80 (1.60) | 3.70 (2.40) | pg/mL | SMD | −0.45 (−1.19 to 0.30) | Reported | Narrative |
| Interferon gamma (IFN-γ) | Vingren et al. (2018) | Final value | 8/8 | 11.40 (12.20) | 10.30 (8.80) | pg/mL | SMD | 0.10 (−0.83 to 1.02) | Reported | Narrative |
| Interleukin-18 (IL-18) | Ryan et al. (2025) | Final value | 15/13 | 143.62 (48.19) | 158.33 (45.63) | ng/mL | SMD | −0.30 (−1.03 to 0.42) | IQR | Narrative |
| Interleukin-2 (IL-2) | Vingren et al. (2018) | Final value | 8/8 | 2.40 (2.00) | 4.60 (7.60) | pg/mL | SMD | −0.37 (−1.31 to 0.56) | Reported | Narrative |
| Interleukin-4 (IL-4) | Pedro et al. (2017) | Final value | 17/11 | 9.70 (6.30) | 13.80 (8.30) | pg/mL | SMD | −0.56 (−1.31 to 0.19) | Reported | Narrative |
| Interleukin-4 (IL-4) | Vingren et al. (2018) | Final value | 8/8 | 14.00 (10.00) | 17.70 (15.80) | pg/mL | SMD | −0.26 (−1.20 to 0.67) | Reported | Narrative |
| Interleukin-5 (IL-5) | Pedro et al. (2017) | Final value | 17/11 | 1.30 (0.60) | 2.40 (2.10) | pg/mL | SMD | −0.77 (−1.53 to −0.01) | Reported | Narrative |
| Irisin | Ghayomzadeh et al. (2022) | Change score | 20/20 | 1.18 (7.87) | 0.16 (3.38) | ng/mL | SMD | 0.17 (−0.44 to 0.77) | Reported | Narrative |
| Leptin | Pedro et al. (2017) | Final value | 12/9 | 8.16 (4.20) | 10.10 (9.50) | pg/mL | SMD | −0.27 (−1.10 to 0.57) | Reported | Narrative |
| Myostatin | Ghayomzadeh et al. (2022) | Change score | 20/20 | −158.90 (62.03) | −43.33 (146.60) | pg/mL | SMD | −1.01 (−1.65 to −0.36) | Reported | Narrative |
| Soluble tumor necrosis factor receptor 2 (sTNFR2) | Dudgeon et al. (2012) | Final value | 16/10 | 2.40 (1.60) | 2.50 (1.58) | ng/mL | SMD | −0.06 (−0.83 to 0.70) | SE | Narrative |
| Testosterone | Dudgeon et al. (2012) | Final value | 16/10 | 65.10 (18.80) | 61.60 (28.14) | pg/mL | SMD | 0.15 (−0.62 to 0.92) | SE | Narrative |
| Vascular cell adhesion molecule 1 (VCAM-1) | Vingren et al. (2018) | Final value | 8/8 | 380.00 (182.00) | 306.00 (223.00) | ng/mL | SMD | 0.34 (−0.59 to 1.28) | Reported | Narrative |

One row per trial, construct and contrast. Form distinguishes final values from change scores. Role records the analysis that each row enters. Primary: the row contributes to the primary analysis of that construct, which for the six inflammatory constructs is the final-value analysis and for CD4+ T-cell count is the single mean-difference analysis that combines final-value and change-score contrasts because both are expressed in cells/mm³. Supporting: the row contributes to the separately synthesized change-score analysis of an inflammatory construct, reported in Table S4, Table S5 and Figure 5. Sensitivity: the row is an alternative factorial-trial contrast used in a post hoc robustness check in Table S4; Primary and Supporting rows may also enter those checks without changing their reference role. Narrative: the construct is reported descriptively without cross-trial pooling, although individual contrasts and confidence intervals may be tabulated. A one-trial Primary or Supporting stratum retains that role and is reported individually, not relabeled Narrative; one-trial Sensitivity results are likewise not pooled. Means, standard deviations and units are reproduced in the units used by the source report and are not converted; standardized effects remove the unit.

SD source records how the standard deviation was obtained: Reported, transcribed directly; SE, derived from a reported standard error as SE × √n; IQR, estimated from a reported median and quartiles using the method of Wan and colleagues; IQR + SE, both methods used across the two arms.

Zanetti et al. (2020) is a 2 × 2 factorial trial reporting change scores only; for the quantitatively synthesized inflammatory constructs it contributes Supporting, not Primary, evidence. The placebo-background contrast enters Supporting analyses; the statin-background and merged-strata contrasts enter separate Sensitivity analyses without double-counting participants, whereas all fibrinogen contrasts remain Narrative. Soares et al. (2022) is a three-arm trial whose two resistance-containing arms were combined into a single group so that the shared control contributes once.

Every value in this table was extracted independently and in duplicate by two reviewers and then checked against the source report by a third reviewer, who compared the numeric value, the group denominator, the unit, the contrast form and the outcome label with the corresponding table, figure or text in the original publication. Zanetti et al. (2020) prints its inflammatory marker units as mg/dL and pg/dL; these are recorded exactly as printed. Because every biomarker construct is pooled as a standardized mean difference, the unit does not enter the pooled estimate.

CI, confidence interval; comp, comparator; CRP, C-reactive protein; IL, interleukin; int, intervention; IQR, interquartile range; MD, mean difference; n, number of participants; SD, standard deviation; SE, standard error; SMD, standardized mean difference; TNF-α, tumor necrosis factor alpha.

**Table S3. Domain-level risk-of-bias judgments with supporting reasoning.**

| **Trial** | **Tool** | **Domain** | **Judgment** | **Reasoning** |
| --- | --- | --- | --- | --- |
| Brito-Neto et al. (2019) | RoB 2 | D1. Randomization process | Some concerns | random allocation was stated, but sequence generation and allocation concealment were not reported. |
| Brito-Neto et al. (2019) | RoB 2 | D2. Deviations from intended interventions | Low | no trial-context deviations from the assigned exercise or no-exercise strategies were evident. |
| Brito-Neto et al. (2019) | RoB 2 | D3. Missing outcome data | Low | the same group denominators (9 and 10) were retained for the CD4 result and no attrition was reported. |
| Brito-Neto et al. (2019) | RoB 2 | D4. Measurement of the outcome | Low | CD4 was measured objectively by flow cytometry. |
| Brito-Neto et al. (2019) | RoB 2 | D5. Selection of the reported result | Some concerns | no prospective protocol or outcome-specific analysis plan was available and several analytical choices were possible. Overall: Some concerns because D1 and D5 were not low. |
| Brito-Neto et al. (2019) | RoB 2 | Overall | Some concerns | Least favorable domain judgment, following the algorithm of the tool applied. |
| Dianatinasab et al. (2018) | RoB 2 | D1. Randomization process | Some concerns | block randomization was reported, but concealment was not described and fixed blocks of size two could be predictable. |
| Dianatinasab et al. (2018) | RoB 2 | D2. Deviations from intended interventions | High | participants were excluded after assignment for non-completion of exercise, so the complete-case analysis did not estimate the effect of assignment. |
| Dianatinasab et al. (2018) | RoB 2 | D3. Missing outcome data | High | 25% of randomized participants lacked post-intervention data, with reasons plausibly related to intervention participation or outcome status. |
| Dianatinasab et al. (2018) | RoB 2 | D4. Measurement of the outcome | Low | CD4 was measured by flow cytometry and laboratory staff were blinded. |
| Dianatinasab et al. (2018) | RoB 2 | D5. Selection of the reported result | Some concerns | no accessible prospective analysis plan was identified and both within-group and between-group analyses were available. Overall: High because D2 and D3 were high. |
| Dianatinasab et al. (2018) | RoB 2 | Overall | High | Least favorable domain judgment, following the algorithm of the tool applied. |
| Dudgeon et al. (2012) | RoB 2 | D1. Randomization process | Some concerns | a random number table was used, but allocation concealment was not reported. |
| Dudgeon et al. (2012) | RoB 2 | D2. Deviations from intended interventions | High | inclusion in the final analysis required completion of at least 10 of 12 sessions, creating a per-protocol analysis that excluded deviations after assignment. |
| Dudgeon et al. (2012) | RoB 2 | D3. Missing outcome data | High | outcome data were available for only a small and inconsistently reported subset of the 111 randomized participants; the biomarker analysis was approximately one quarter of the randomized cohort. |
| Dudgeon et al. (2012) | RoB 2 | D4. Measurement of the outcome | Low | cytokines and hormones were measured with standardized immunoassays/ELISAs. |
| Dudgeon et al. (2012) | RoB 2 | D5. Selection of the reported result | Some concerns | no prospective outcome-specific analysis plan was available, and multiple eligible time points, sample matrices, and post hoc body-fat subgroup analyses were used. Overall: High because D2 and D3 were high. |
| Dudgeon et al. (2012) | RoB 2 | Overall | High | Least favorable domain judgment, following the algorithm of the tool applied. |
| Ghayomzadeh et al. (2019) | RoB 2 | D1. Randomization process | Some concerns | random assignment was stated, but the sequence method and concealment were not reported. |
| Ghayomzadeh et al. (2019) | RoB 2 | D2. Deviations from intended interventions | Low | no important deviations from assigned intervention were identified. |
| Ghayomzadeh et al. (2019) | RoB 2 | D3. Missing outcome data | Some concerns | 3/21 participants (14%) were missing and reasons were not reported; the balanced direction reduces but does not remove concern. |
| Ghayomzadeh et al. (2019) | RoB 2 | D4. Measurement of the outcome | Low | CD4 was measured objectively in a licensed clinical laboratory. |
| Ghayomzadeh et al. (2019) | RoB 2 | D5. Selection of the reported result | Low | the trial was registered, CD4 was identified as the primary outcome, and no evidence of selective non-reporting of that outcome was found. Overall: Some concerns because D1 and D3 were not low. |
| Ghayomzadeh et al. (2019) | RoB 2 | Overall | Some concerns | Least favorable domain judgment, following the algorithm of the tool applied. |
| Ghayomzadeh et al. (2022) | RoB 2 | D1. Randomization process | Some concerns | equal random allocation was reported, but sequence generation and allocation concealment were not described. |
| Ghayomzadeh et al. (2022) | RoB 2 | D2. Deviations from intended interventions | Low | supervised training and usual-care control were implemented without evidence of trial-context deviations affecting outcomes. |
| Ghayomzadeh et al. (2022) | RoB 2 | D3. Missing outcome data | Low | only 3/40 participants were missing, losses were balanced and documented for reasons unlikely to depend on biomarker values. |
| Ghayomzadeh et al. (2022) | RoB 2 | D4. Measurement of the outcome | Low | outcomes were measured using standardized laboratory assays by blinded assessors/technicians. |
| Ghayomzadeh et al. (2022) | RoB 2 | D5. Selection of the reported result | Low | the trial was registered and the companion reports specified the relevant secondary outcomes; no evidence of selective omission among the extracted outcomes was identified. Overall: Some concerns because D1 was not low. |
| Ghayomzadeh et al. (2022) | RoB 2 | Overall | Some concerns | Least favorable domain judgment, following the algorithm of the tool applied. |
| Gomes et al. (2010) | ROBINS-I | D1. Confounding | Serious | intervention uptake was self-selected and no adequate adjustment for prognostic differences was performed. |
| Gomes et al. (2010) | ROBINS-I | D2. Selection of participants | Serious | control membership depended partly on post-enrollment attendance/dropout and waiting-list status, both related to intervention participation. |
| Gomes et al. (2010) | ROBINS-I | D3. Classification of interventions | Low | intervention exposure was clearly distinguishable despite the mixed control composition. |
| Gomes et al. (2010) | ROBINS-I | D4. Deviations from intended interventions | Moderate | some controls had partial exercise exposure and co-intervention/contamination control was incompletely described. |
| Gomes et al. (2010) | ROBINS-I | D5. Missing data | Low | no dropouts occurred during the 12-week study after groups were formed. |
| Gomes et al. (2010) | ROBINS-I | D6. Measurement of outcomes | Low | CD4 was objectively measured by flow cytometry and examiners were blinded. |
| Gomes et al. (2010) | ROBINS-I | D7. Selection of the reported result | Moderate | no prespecified analysis plan was available and multiple outcome representations/analyses were possible. Overall: Serious because confounding and participant selection were serious. |
| Gomes et al. (2010) | ROBINS-I | Overall | Serious | Least favorable domain judgment, following the algorithm of the tool applied. |
| Khoramipour et al. (2022) | RoB 2 | D1. Randomization process | High | even/odd allocation was predictable and therefore did not provide concealed random allocation. |
| Khoramipour et al. (2022) | RoB 2 | D2. Deviations from intended interventions | High | the analysis excluded participants after assignment according to attendance, so it did not estimate the effect of assignment. |
| Khoramipour et al. (2022) | RoB 2 | D3. Missing outcome data | Some concerns | 4/30 outcomes were missing; losses were balanced, but were related to adherence and may be prognostic. |
| Khoramipour et al. (2022) | RoB 2 | D4. Measurement of the outcome | Low | the outcomes were objectively measured using laboratory assays with standardized timing. |
| Khoramipour et al. (2022) | RoB 2 | D5. Selection of the reported result | Some concerns | no prospective outcome-specific analysis plan was available and multiple outcome/analysis choices were possible. Overall: High because D1 and D2 were high. |
| Khoramipour et al. (2022) | RoB 2 | Overall | High | Least favorable domain judgment, following the algorithm of the tool applied. |
| Pedro et al. (2017) | RoB 2 | D1. Randomization process | Low | a random-number sequence and sequential opaque sealed envelopes were reported, with no evident baseline imbalance. |
| Pedro et al. (2017) | RoB 2 | D2. Deviations from intended interventions | Low | no systematic deviations from the assigned strategies were demonstrated; attrition is addressed in D3. |
| Pedro et al. (2017) | RoB 2 | D3. Missing outcome data | High | more than half of randomized participants lacked analyzed cytokine data, and the companion report used an even smaller biomarker subset with incomplete reconciliation of denominators and reasons. |
| Pedro et al. (2017) | RoB 2 | D4. Measurement of the outcome | Low | objective laboratory methods were used and outcome assessors were blinded. |
| Pedro et al. (2017) | RoB 2 | D5. Selection of the reported result | Some concerns | no accessible prospective outcome-specific analysis plan was identified, and the trial generated multiple cytokines, companion reports, repeated-measures analyses and magnitude-based analyses. Overall: High because D3 was high. |
| Pedro et al. (2017) | RoB 2 | Overall | High | Least favorable domain judgment, following the algorithm of the tool applied. |
| Piraux et al. (2019) | RoB 2 | D1. Randomization process | Some concerns | computer-generated randomization was reported, but allocation concealment was not described. |
| Piraux et al. (2019) | RoB 2 | D2. Deviations from intended interventions | Low | no important trial-context deviations from assignment were identified. |
| Piraux et al. (2019) | RoB 2 | D3. Missing outcome data | High | 8/25 participants (32%) lacked outcome data, and reasons such as deterioration, motivation and unknown causes could relate to true outcomes. |
| Piraux et al. (2019) | RoB 2 | D4. Measurement of the outcome | Low | CRP, D-dimer, CD4 and viral load were objective blood measurements. |
| Piraux et al. (2019) | RoB 2 | D5. Selection of the reported result | Low | the pilot trial was prospectively registered, feasibility was clearly primary, secondary outcomes and the multiplicity correction were reported, and no selective omission of the extracted outcomes was evident. Overall: High because D3 was high. |
| Piraux et al. (2019) | RoB 2 | Overall | High | Least favorable domain judgment, following the algorithm of the tool applied. |
| Ryan et al. (2025) | RoB 2 | D1. Randomization process | Some concerns | 1:1 random allocation was stated, but sequence generation and allocation concealment were not reported. |
| Ryan et al. (2025) | RoB 2 | D2. Deviations from intended interventions | Low | supervised exercise and sedentary control were clearly specified with no evident differential deviation. |
| Ryan et al. (2025) | RoB 2 | D3. Missing outcome data | Some concerns | 5/33 participants (15%) lacked final data and outcome-specific reasons were not fully reported in text. |
| Ryan et al. (2025) | RoB 2 | D4. Measurement of the outcome | Low | biomarkers were measured objectively in duplicate with standardized assays. |
| Ryan et al. (2025) | RoB 2 | D5. Selection of the reported result | Some concerns | no accessible outcome-specific analysis plan was identified, and numerous between-group, within-group, percent-change, correlation and post hoc power analyses were available. Overall: Some concerns because D1, D3 and D5 were not low. |
| Ryan et al. (2025) | RoB 2 | Overall | Some concerns | Least favorable domain judgment, following the algorithm of the tool applied. |
| Soares et al. (2022) | RoB 2 | D1. Randomization process | Some concerns | a computerized sequence was used, but allocation concealment was not reported. |
| Soares et al. (2022) | RoB 2 | D2. Deviations from intended interventions | Low | supervised protocols and the non-exercise control were implemented without evidence of differential deviations. |
| Soares et al. (2022) | RoB 2 | D3. Missing outcome data | Low | all 41 allocated participants were represented in the outcome tables, with no reported outcome attrition. |
| Soares et al. (2022) | RoB 2 | D4. Measurement of the outcome | Low | investigators were blinded during collection and immune outcomes were objectively assayed. |
| Soares et al. (2022) | RoB 2 | D5. Selection of the reported result | Low | the trial was registered and the reported immune outcomes matched the stated assessments, with no evidence of selective result omission. Overall: Some concerns because D1 was not low. |
| Soares et al. (2022) | RoB 2 | Overall | Some concerns | Least favorable domain judgment, following the algorithm of the tool applied. |
| Vingren et al. (2018) | RoB 2 | D1. Randomization process | Some concerns | random assignment was stated, but sequence generation and allocation concealment were not described. |
| Vingren et al. (2018) | RoB 2 | D2. Deviations from intended interventions | Low | both groups received the same inpatient standard care and no differential deviations from assigned exercise strategy were reported. |
| Vingren et al. (2018) | RoB 2 | D3. Missing outcome data | Low | target biomarker data were available for the small randomized cohort; missing baseline drug-history information did not constitute missing outcome data. |
| Vingren et al. (2018) | RoB 2 | D4. Measurement of the outcome | Low | fasting samples were standardized and biomarkers were measured objectively in duplicate. |
| Vingren et al. (2018) | RoB 2 | D5. Selection of the reported result | Some concerns | no prospective outcome-specific analysis plan was available for the broad cytokine panel and multiple analyses were possible. Overall: Some concerns because D1 and D5 were not low. |
| Vingren et al. (2018) | RoB 2 | Overall | Some concerns | Least favorable domain judgment, following the algorithm of the tool applied. |
| Yarasheski et al. (2011) | RoB 2 | D1. Randomization process | Some concerns | random assignment was reported, but sequence generation and allocation concealment were not described. |
| Yarasheski et al. (2011) | RoB 2 | D2. Deviations from intended interventions | Low | both groups received pioglitazone and the exercise contrast was clearly implemented; no major differential deviations were documented. |
| Yarasheski et al. (2011) | RoB 2 | D3. Missing outcome data | Some concerns | 5/44 participants (11%) lacked final data and several losses were related to noncompliance, which could be prognostic. |
| Yarasheski et al. (2011) | RoB 2 | D4. Measurement of the outcome | Low | adiponectin was measured objectively by a commercial ELISA. |
| Yarasheski et al. (2011) | RoB 2 | D5. Selection of the reported result | Low | the trial was registered and primary/secondary metabolic outcomes were stated, with no evidence that the adiponectin result was selectively chosen. Overall: Some concerns because D1 and D3 were not low. |
| Yarasheski et al. (2011) | RoB 2 | Overall | Some concerns | Least favorable domain judgment, following the algorithm of the tool applied. |
| Zanetti et al. (2016) | RoB 2 | D1. Randomization process | Some concerns | random allocation was stated, but the sequence method and concealment were not described. |
| Zanetti et al. (2016) | RoB 2 | D2. Deviations from intended interventions | Low | the intervention was supervised, attendance was 100%, and the control maintained usual activities. |
| Zanetti et al. (2016) | RoB 2 | D3. Missing outcome data | Low | all 30 participants were retained in the reported outcome analyses. |
| Zanetti et al. (2016) | RoB 2 | D4. Measurement of the outcome | Low | blinded assessment and objective flow-cytometric/colorimetric methods were used. |
| Zanetti et al. (2016) | RoB 2 | D5. Selection of the reported result | Low | the registered companion reports together presented the prespecified inflammatory, immune and CRP outcomes with no evidence of selective omission. Overall: Some concerns because D1 was not low. |
| Zanetti et al. (2016) | RoB 2 | Overall | Some concerns | Least favorable domain judgment, following the algorithm of the tool applied. |
| Zanetti et al. (2020) | RoB 2 | D1. Randomization process | Some concerns | computerized randomization and blinding procedures were reported, but concealment at enrollment was not explicitly described. |
| Zanetti et al. (2020) | RoB 2 | D2. Deviations from intended interventions | Low | exercise and medication/placebo strategies were prespecified and no important differential deviation was evident. |
| Zanetti et al. (2020) | RoB 2 | D3. Missing outcome data | Some concerns | 11/83 participants (13.25%) were missing, including five withdrawals without reasons; this could plausibly bias a small factorial contrast. |
| Zanetti et al. (2020) | RoB 2 | D4. Measurement of the outcome | Low | evaluators were blinded and biomarkers were objectively assayed. |
| Zanetti et al. (2020) | RoB 2 | D5. Selection of the reported result | Low | the trial was registered and the reported inflammatory outcomes matched the stated outcome set, with no evidence of selective omission. Overall: Some concerns because D1 and D3 were not low. |
| Zanetti et al. (2020) | RoB 2 | Overall | Some concerns | Least favorable domain judgment, following the algorithm of the tool applied. |

Judgments are reported as text without color coding; the same grid is presented graphically elsewhere and is not reproduced here as a colored matrix.

Risk of bias was assessed by one reviewer, and every domain-level judgment together with its supporting reasoning was checked against the source report by a second reviewer; queries raised during checking were resolved by discussion and by returning to the source report. The judgments were not made independently in duplicate, as stated among the limitations in Section 4.5 of the main text. The judgments presented here relate to the eligible biomarker and T-cell outcomes contributed by each trial and are reported once per trial, with the supporting reasoning reproduced so that each judgment can be checked against its source. RoB 2 was applied to the randomized and quasi-randomized trials and yields five domains. ROBINS-I was applied to the single non-randomized controlled trial and yields seven domains.

RoB 2, revised Cochrane risk-of-bias tool for randomized trials; ROBINS-I, Risk Of Bias In Non-randomized Studies of Interventions.

**Table S4. Sensitivity analyses with Primary and Supporting reference estimates.**

| **Outcome** | **Analysis** | **Question addressed** | **Trials, k** | **Effect (95% CI)** | **I², %** |
| --- | --- | --- | --- | --- | --- |
| Interleukin-6 (IL-6) | Primary final-value analysis | Reference analysis for the inflammatory construct. | 4 | −0.19 (−1.14 to 0.76) | 48.5 |
| Interleukin-6 (IL-6) | Common-effect model (final values) | Does the variance model alter the primary conclusion? | 4 | −0.22 (−0.61 to 0.18) | 48.5 |
| Interleukin-6 (IL-6) | Excluding the recreationally active comparator | Does excluding the recreationally active comparator alter the final-value estimate? | 3 | −0.14 (−2.00 to 1.73) | 65.5 |
| Interleukin-6 (IL-6) | Final-value trials with directly reported SDs | Do reconstructed dispersion values drive the final-value estimate? | 2 | 0.18 (−6.13 to 6.48) | 61.5 |
| Interleukin-6 (IL-6) | Supporting change-score analysis | What does the separately synthesized change-score evidence show? | 3 | −3.32 (−10.85 to 4.21) | 96.1 |
| Interleukin-6 (IL-6) | Change scores: statin-background stratum | Does the alternative handling of the factorial trial alter the change-score estimate? | 3 | −3.67 (−9.93 to 2.59) | 92.7 |
| Interleukin-6 (IL-6) | Change scores: both medication strata merged | Does the alternative handling of the factorial trial alter the change-score estimate? | 3 | −3.46 (−10.45 to 3.53) | 95.8 |
| Tumor necrosis factor alpha (TNF-α) | Primary final-value analysis | Reference analysis for the inflammatory construct. | 3 | 0.07 (−1.44 to 1.57) | 49.9 |
| Tumor necrosis factor alpha (TNF-α) | Common-effect model (final values) | Does the variance model alter the primary conclusion? | 3 | 0.02 (−0.44 to 0.47) | 49.9 |
| Tumor necrosis factor alpha (TNF-α) | Excluding the recreationally active comparator | Does excluding the recreationally active comparator alter the final-value estimate? | 2 | 0.34 (−5.12 to 5.79) | 49.5 |
| Tumor necrosis factor alpha (TNF-α) | Final-value trials with directly reported SDs | Do reconstructed dispersion values drive the final-value estimate? | 2 | 0.17 (−7.70 to 8.04) | 74.7 |
| Tumor necrosis factor alpha (TNF-α) | Supporting change-score analysis | What does the separately synthesized change-score evidence show? | 2 | −3.03 (−7.44 to 1.38) | 0.0 |
| C-reactive protein (CRP) | Primary final-value analysis | Reference analysis for the inflammatory construct. | 3 | −0.02 (−1.46 to 1.41) | 46.4 |
| C-reactive protein (CRP) | Common-effect model (final values) | Does the variance model alter the primary conclusion? | 3 | −0.06 (−0.53 to 0.41) | 46.4 |
| C-reactive protein (CRP) | Excluding the recreationally active comparator | Does excluding the recreationally active comparator alter the final-value estimate? | 2 | −0.13 (−6.67 to 6.40) | 66.1 |
| C-reactive protein (CRP) | Final-value trials with directly reported SDs | Do reconstructed dispersion values drive the final-value estimate? | 1 | Individual result: 0.25 (−0.58 to 1.08) | — |
| C-reactive protein (CRP) | Supporting change-score analysis | What does the separately synthesized change-score evidence show? | 1 | Individual result: −1.66 (−2.40 to −0.91) | — |
| C-reactive protein (CRP) | Change scores: statin-background stratum | Does the alternative handling of the factorial trial alter the change-score estimate? | 1 | Individual result: −0.02 (−0.66 to 0.62) | — |
| C-reactive protein (CRP) | Change scores: both medication strata merged | Does the alternative handling of the factorial trial alter the change-score estimate? | 1 | Individual result: −0.60 (−1.07 to −0.13) | — |
| Interleukin-1 beta (IL-1β) | Primary final-value analysis | Reference analysis for the inflammatory construct. | 2 | −0.47 (−10.09 to 9.15) | 81.4 |
| Interleukin-1 beta (IL-1β) | Common-effect model (final values) | Does the variance model alter the primary conclusion? | 2 | −0.30 (−0.91 to 0.32) | 81.4 |
| Interleukin-1 beta (IL-1β) | Final-value trials with directly reported SDs | Do reconstructed dispersion values drive the final-value estimate? | 1 | Individual result: −1.27 (−2.29 to −0.24) | — |
| Interleukin-1 beta (IL-1β) | Supporting change-score analysis | What does the separately synthesized change-score evidence show? | 2 | −2.01 (−5.97 to 1.94) | 7.5 |
| Interleukin-1 beta (IL-1β) | Change scores: statin-background stratum | Does the alternative handling of the factorial trial alter the change-score estimate? | 2 | −1.79 (−8.86 to 5.28) | 71.9 |
| Interleukin-1 beta (IL-1β) | Change scores: both medication strata merged | Does the alternative handling of the factorial trial alter the change-score estimate? | 2 | −1.74 (−9.08 to 5.61) | 78.9 |
| Interleukin-8 (IL-8) | Primary final-value analysis | Reference analysis for the inflammatory construct. | 1 | Individual result: −0.63 (−1.39 to 0.12) | — |
| Interleukin-8 (IL-8) | Supporting change-score analysis | What does the separately synthesized change-score evidence show? | 2 | −1.16 (−8.85 to 6.53) | 80.2 |
| Interleukin-8 (IL-8) | Change scores: statin-background stratum | Does the alternative handling of the factorial trial alter the change-score estimate? | 2 | −1.02 (−10.58 to 8.55) | 87.3 |
| Interleukin-8 (IL-8) | Change scores: both medication strata merged | Does the alternative handling of the factorial trial alter the change-score estimate? | 2 | −1.04 (−10.09 to 8.02) | 88.5 |
| Interleukin-10 (IL-10) | Primary final-value analysis | Reference analysis for the inflammatory construct. | 2 | 0.04 (−11.37 to 11.46) | 87.4 |
| Interleukin-10 (IL-10) | Common-effect model (final values) | Does the variance model alter the primary conclusion? | 2 | −0.15 (−0.75 to 0.46) | 87.4 |
| Interleukin-10 (IL-10) | Excluding the recreationally active comparator | Does excluding the recreationally active comparator alter the final-value estimate? | 1 | Individual result: 0.97 (−0.02 to 1.96) | — |
| Interleukin-10 (IL-10) | Supporting change-score analysis | What does the separately synthesized change-score evidence show? | 2 | 2.37 (−10.58 to 15.32) | 89.0 |
| Interleukin-10 (IL-10) | Change scores: statin-background stratum | Does the alternative handling of the factorial trial alter the change-score estimate? | 2 | 2.21 (−12.86 to 17.28) | 92.1 |
| Interleukin-10 (IL-10) | Change scores: both medication strata merged | Does the alternative handling of the factorial trial alter the change-score estimate? | 2 | 2.27 (−11.74 to 16.29) | 92.1 |
| CD4+ T-cell count | Primary analysis (random effects) | Reference analysis for comparison. | 7 | 124.2 (54.6 to 193.7) | 71.3 |
| CD4+ T-cell count | Common-effect model | Does the variance model alter the conclusion? | 7 | 106.1 (83.2 to 129.0) | 71.3 |
| CD4+ T-cell count | Randomized trials only | Does the non-randomized trial drive the estimate? | 6 | 114.0 (28.2 to 199.8) | 66.2 |
| CD4+ T-cell count | Final-value contrasts only | What is the estimate among final-value contrasts? | 5 | 140.5 (34.7 to 246.3) | 56.1 |
| CD4+ T-cell count | Change-score contrasts only | What is the estimate among change-score contrasts? | 2 | 100.8 (−495.5 to 697.2) | 82.9 |
| CD4+ T-cell count | Excluding the trial with marked baseline imbalance | Does the trial with a large baseline CD4 difference drive the estimate? | 6 | 111.2 (50.9 to 171.6) | 67.2 |
| CD4+ T-cell count | Treating the flagged dispersion as a standard error | Does treating the flagged dispersion as an SEM alter the estimate? | 7 | 116.4 (44.3 to 188.6) | 60.9 |

Primary and Supporting rows are reference estimates, not Sensitivity analyses; the remaining rows are post hoc exploratory robustness checks. For inflammatory biomarkers, the primary analysis uses final-value standardized mean differences; change-score standardized mean differences are synthesized separately as supporting evidence. Rows containing one trial are individual study results and are not pooled. Checks that would leave an analysis unchanged are omitted.

The common-effect check replaces the random-effects model only to show the influence of the variance model. It is not an alternative primary analysis. Alternative statin-background and merged-stratum analyses are Sensitivity checks of the Supporting inflammatory change-score evidence; the CD4+ final-value-only and change-score-only analyses are Sensitivity checks of its combined-form Primary analysis.

CI, confidence interval; I², percentage of variability across trials attributable to heterogeneity rather than chance; k, number of trials; MD, mean difference; SD, standard deviation; SEM, standard error of the mean; SMD, standardized mean difference. I² is omitted when k = 1.

**Table S5. Post hoc subgroup analyses, separate contrast-form analyses, and screening of candidate moderators.**

**Panel A. Subgroup analyses for CD4+ T-cell count**

| **Moderator** | **Level** | **Trials, k** | **Effect (95% CI)** | **I², %** | **P for difference** |
| --- | --- | --- | --- | --- | --- |
| Intervention type | Resistance training only | 3 | 135.8 (−160.5 to 432.1) | 80 | 0.837 |
| Intervention type | Resistance-containing combined training | 4 | 150.7 (79.6 to 221.8) | 17 | 0.837 |
| Overall risk of bias | High or serious | 3 | 121.0 (−97.2 to 339.2) | NR | 0.893 |
| Overall risk of bias | Some concerns | 4 | 130.1 (−10.6 to 270.7) | NR | 0.893 |

Panel B. Final-value and change-score estimates reported separately

| **Outcome** | **Final-value estimate** | **Change-score estimate** | **Analysis status** | **Formal between-form inference** |
| --- | --- | --- | --- | --- |
| Interleukin-6 (IL-6) | 4; −0.19 (−1.14 to 0.76) | 3; −3.32 (−10.85 to 4.21) | Both pooled | Not performed; post hoc and confounded |
| Tumor necrosis factor alpha (TNF-α) | 3; 0.07 (−1.44 to 1.57) | 2; −3.03 (−7.44 to 1.38) | Both pooled | Not performed; post hoc and confounded |
| C-reactive protein (CRP) | 3; −0.02 (−1.46 to 1.41) | 1; −1.66 (−2.40 to −0.91) | Final-value pooled; change-score individual | Not performed; post hoc and confounded |
| Interleukin-1 beta (IL-1β) | 2; −0.47 (−10.09 to 9.15) | 2; −2.01 (−5.97 to 1.94) | Both pooled | Not performed; post hoc and confounded |
| Interleukin-8 (IL-8) | 1; −0.63 (−1.39 to 0.12) | 2; −1.16 (−8.85 to 6.53) | Final-value individual; change-score pooled | Not performed; post hoc and confounded |
| Interleukin-10 (IL-10) | 2; 0.04 (−11.37 to 11.46) | 2; 2.37 (−10.58 to 15.32) | Both pooled | Not performed; post hoc and confounded |
| CD4+ T-cell count | 5; 140.5 (34.7 to 246.3) | 2; 100.8 (−495.5 to 697.2) | Both pooled; Sensitivity analyses | Descriptive only; Primary MD analysis combines forms |

**Panel C. Screening of candidate moderator variables**

| **Variable** | **Type** | **Decision** | **Levels applied** | **Reason** |
| --- | --- | --- | --- | --- |
| Intervention type | Categorical | Retained for subgroup analysis | Resistance training only \| Resistance-containing combined training | Clinically important and met the entry condition of at least three trials in every level for CD4+ T-cell count. |
| Overall risk of bias | Ordinal | Retained for subgroup analysis | Some concerns \| High or serious | Met the entry condition of at least three trials in every level for CD4+ T-cell count and supports interpretation of certainty. |
| Intervention duration, weeks | Dose | Not analyzed | <12 weeks \| ≥12 weeks | Only two CD4+ trials lasted <12 weeks; the condition of at least three trials in every level was not met. |
| Data form | Categorical | Defines inflammatory Primary/Supporting analyses; CD4+ subsets are Sensitivity analyses | Final values \| Change from baseline | Final-value and change-score SMDs use different standardizing dispersions and are reported separately. This analytic decision was post hoc; no formal moderator inference was made. |
| Publication year | Mixed | Not analyzed | — | Continuous covariate; meta-regression is not interpretable with this few trials. |
| Mean age, years | Mixed | Not analyzed | — | Continuous covariate; meta-regression is not interpretable with this few trials. |
| Female participants, % | Mixed | Not analyzed | — | Continuous covariate; meta-regression is not interpretable with this few trials. |
| Attrition, % | Mixed | Not analyzed | — | Continuous covariate; meta-regression is not interpretable with this few trials. |
| Geographic region | Mixed | Not analyzed | — | Four or more levels with one to three trials each; no level is separable. |
| Blood-draw timing category | Mixed | Not analyzed | — | Timing relative to the last session is not reported by six trials. |
| Baseline CD4 status | Mixed | Not analyzed | — | Reported criteria are heterogeneous and frequently absent. |
| Special population | Mixed | Not analyzed | — | Each flagged population is represented by a single trial. |
| Supervision mode | Mixed | Not analyzed | — | Almost all trials delivered supervised training; no contrast exists. |
| Total resistance-training volume, kg | Mixed | Not analyzed | — | Reported by one trial only. |
| Exercise adherence, % | Mixed | Not analyzed | — | Reported by too few trials to support comparison. |
| Resistance intensity, % 1RM midpoint | Mixed | Not analyzed | — | Prescriptions are expressed on noncomparable scales (percentage 1RM, repetition-maximum zones, or perceived exertion). |

All subgroup analyses and moderator screening were post hoc exploratory. Subgroup analysis was restricted to clinically interpretable moderators with at least three trials in every level and was possible only for CD4+ T-cell count. No meta-regression was fitted because the number of trials per construct was too small for a stable slope.

Panel B does not contain a mixed-form standardized mean difference. For each inflammatory construct, final-value standardized effects are the primary evidence and change-score standardized effects are supporting evidence. A stratum with at least two trials is pooled; a one-trial stratum is identified as an individual result. The rows are compared descriptively only. No between-form test was performed; the small, confounded strata cannot establish an independent causal or statistical contribution to heterogeneity. Both CD4+ columns are Sensitivity analyses of the combined-form Primary mean difference in cells/mm³, not separate Primary or Supporting analyses.

CI, confidence interval; k, number of trials; MD, mean difference; NR, not reported; 1RM, one-repetition maximum; RoB, risk of bias; SMD, standardized mean difference.

**Table S6. GRADE certainty of evidence, by construct and domain.**

**Panel A. Construct-level GRADE ratings**

| **Outcome** | **Trials (participants)** | **Start** | **Risk of bias** | **Inconsistency** | **Indirectness** | **Imprecision** | **Publication bias** | **Levels down** | **Certainty** |
| --- | --- | --- | --- | --- | --- | --- | --- | --- | --- |
| Interleukin-6 (IL-6) | 4 (98) | High | Serious | Serious | Not serious | Serious | Not assessed | 3 | Very low |
| Tumor necrosis factor alpha (TNF-α) | 3 (72) | High | Serious | Serious | Not serious | Serious | Not assessed | 3 | Very low |
| C-reactive protein (CRP) | 3 (66) | High | Serious | Serious | Not serious | Serious | Not assessed | 3 | Very low |
| Interleukin-1 beta (IL-1β) | 2 (42) | High | Serious | Serious | Not serious | Serious | Not assessed | 3 | Very low |
| Interleukin-8 (IL-8) | 1 (28) | High | Serious | Not estimable (one trial; no downgrade) | Not serious | Very serious | Not assessed | 3 | Very low |
| Interleukin-10 (IL-10) | 2 (44) | High | Serious | Serious | Not serious | Serious | Not assessed | 3 | Very low |
| CD4+ T-cell count | 7 (184) | High | Serious | Serious | Not serious | Serious | Not assessed | 3 | Very low |

**Panel B. Domain-by-domain reasoning**

| **Outcome** | **Domain** | **Rating** | **Reasoning** |
| --- | --- | --- | --- |
| Interleukin-6 (IL-6) | Risk of bias | Serious | No contributing trial was at overall low risk of bias; 2 of 4 trial(s) were at high or serious risk and carried 52% of the random-effects weight. Allocation concealment was generally unclear. |
| Interleukin-6 (IL-6) | Inconsistency | Serious | I² = 48% and the prediction interval (−2.29 to 1.91) spans effects in both directions. With only 4 trials, sources of variation cannot be separated reliably. |
| Interleukin-6 (IL-6) | Indirectness | Not serious | All comparators met the prespecified structured-exercise versus no-prescribed-resistance-program contrast. Placebo, medication, or care backgrounds were balanced between arms and therefore did not make the exercise contrast indirect; the recreationally active comparator was examined in robustness analysis. |
| Interleukin-6 (IL-6) | Imprecision | Serious | The confidence interval (−1.14 to 0.76) is wide and only 98 participants contribute, far below a conventional optimal information size. |
| Interleukin-6 (IL-6) | Publication bias | Not assessed | Not assessed by funnel-plot methods because only 4 trial(s) contribute, below the usual minimum of 10. Small-study effects cannot be excluded; no additional downgrade was applied. |
| Tumor necrosis factor alpha (TNF-α) | Risk of bias | Serious | No contributing trial was at overall low risk of bias; 1 of 3 trial(s) were at high or serious risk and carried 36% of the random-effects weight. Allocation concealment was generally unclear. |
| Tumor necrosis factor alpha (TNF-α) | Inconsistency | Serious | I² = 50% and the prediction interval (−6.75 to 6.89) spans effects in both directions. With only 3 trials, sources of variation cannot be separated reliably. |
| Tumor necrosis factor alpha (TNF-α) | Indirectness | Not serious | All comparators met the prespecified structured-exercise versus no-prescribed-resistance-program contrast. Placebo, medication, or care backgrounds were balanced between arms and therefore did not make the exercise contrast indirect; the recreationally active comparator was examined in robustness analysis. |
| Tumor necrosis factor alpha (TNF-α) | Imprecision | Serious | The confidence interval (−1.44 to 1.57) is wide and only 72 participants contribute, far below a conventional optimal information size. |
| Tumor necrosis factor alpha (TNF-α) | Publication bias | Not assessed | Not assessed by funnel-plot methods because only 3 trial(s) contribute, below the usual minimum of 10. Small-study effects cannot be excluded; no additional downgrade was applied. |
| C-reactive protein (CRP) | Risk of bias | Serious | No contributing trial was at overall low risk of bias; 2 of 3 trial(s) were at high or serious risk and carried 63% of the random-effects weight. Allocation concealment was generally unclear. |
| C-reactive protein (CRP) | Inconsistency | Serious | I² = 46% and the prediction interval (−6.58 to 6.54) spans effects in both directions. With only 3 trials, sources of variation cannot be separated reliably. |
| C-reactive protein (CRP) | Indirectness | Not serious | All comparators met the prespecified structured-exercise versus no-prescribed-resistance-program contrast. Placebo, medication, or care backgrounds were balanced between arms and therefore did not make the exercise contrast indirect; the recreationally active comparator was examined in robustness analysis. |
| C-reactive protein (CRP) | Imprecision | Serious | The confidence interval (−1.46 to 1.41) is wide and only 66 participants contribute, far below a conventional optimal information size. |
| C-reactive protein (CRP) | Publication bias | Not assessed | Not assessed by funnel-plot methods because only 3 trial(s) contribute, below the usual minimum of 10. Small-study effects cannot be excluded; no additional downgrade was applied. |
| Interleukin-1 beta (IL-1β) | Risk of bias | Serious | No contributing trial was at overall low risk of bias; 1 of 2 trial(s) were at high or serious risk and carried 53% of the random-effects weight. Allocation concealment was generally unclear. |
| Interleukin-1 beta (IL-1β) | Inconsistency | Serious | I² = 81% across only 2 trials, with materially different trial estimates and insufficient information to explain the variation. |
| Interleukin-1 beta (IL-1β) | Indirectness | Not serious | All comparators met the prespecified structured-exercise versus no-prescribed-resistance-program contrast. Shared care backgrounds did not make the exercise contrast indirect; no recreationally active comparator contributed to this final-value analysis. |
| Interleukin-1 beta (IL-1β) | Imprecision | Serious | The confidence interval (−10.09 to 9.15) is wide and only 42 participants contribute, far below a conventional optimal information size. |
| Interleukin-1 beta (IL-1β) | Publication bias | Not assessed | Not assessed by funnel-plot methods because only 2 trial(s) contribute, below the usual minimum of 10. Small-study effects cannot be excluded; no additional downgrade was applied. |
| Interleukin-8 (IL-8) | Risk of bias | Serious | The single contributing trial, Pedro et al. (2017), was at high overall risk because more than half of randomized participants lacked analyzed cytokine data. This is an individual study result, not a random-effects meta-analysis. |
| Interleukin-8 (IL-8) | Inconsistency | Not estimable | Only one final-value trial contributes, so between-trial inconsistency cannot be estimated; no downgrade was applied in this domain. |
| Interleukin-8 (IL-8) | Indirectness | Not serious | All comparators met the prespecified structured-exercise versus no-prescribed-resistance-program contrast. The recreationally active comparator met eligibility; removing this sole trial would leave no final-value evidence, so no comparator-exclusion analysis was possible. |
| Interleukin-8 (IL-8) | Imprecision | Very serious | One small trial with 28 participants yielded a confidence interval spanning a potentially important reduction and no effect; imprecision was downgraded by two levels. |
| Interleukin-8 (IL-8) | Publication bias | Not assessed | Not assessed by funnel-plot methods because only 1 trial(s) contribute, below the usual minimum of 10. Small-study effects cannot be excluded; no additional downgrade was applied. |
| Interleukin-10 (IL-10) | Risk of bias | Serious | No contributing trial was at overall low risk of bias; 1 of 2 trial(s) were at high or serious risk and carried 52% of the random-effects weight. Allocation concealment was generally unclear. |
| Interleukin-10 (IL-10) | Inconsistency | Serious | I² = 87% across only 2 trials, with materially different trial estimates and insufficient information to explain the variation. |
| Interleukin-10 (IL-10) | Indirectness | Not serious | All comparators met the prespecified structured-exercise versus no-prescribed-resistance-program contrast. Placebo, medication, or care backgrounds were balanced between arms and therefore did not make the exercise contrast indirect; the recreationally active comparator was examined in robustness analysis. |
| Interleukin-10 (IL-10) | Imprecision | Serious | The confidence interval (−11.37 to 11.46) is wide and only 44 participants contribute, far below a conventional optimal information size. |
| Interleukin-10 (IL-10) | Publication bias | Not assessed | Not assessed by funnel-plot methods because only 2 trial(s) contribute, below the usual minimum of 10. Small-study effects cannot be excluded; no additional downgrade was applied. |
| CD4+ T-cell count | Risk of bias | Serious | No contributing trial was at overall low risk of bias; 3 of 7 trial(s) were at high or serious risk and carried 32% of the random-effects weight. Allocation concealment was generally unclear. |
| CD4+ T-cell count | Inconsistency | Serious | I² = 71% and the prediction interval (−27.05 to 275.42) spans effects in both directions. With only 7 trials, sources of variation cannot be separated reliably. |
| CD4+ T-cell count | Indirectness | Not serious | All comparators met the prespecified structured-exercise versus no-prescribed-resistance-program contrast. No recreationally active comparator contributed to this analysis; confounding in the non-randomized trial was addressed in risk of bias and in the randomized-only Sensitivity analysis. |
| CD4+ T-cell count | Imprecision | Serious | The confidence interval (54.62 to 193.75) is wide and only 184 participants contribute, far below a conventional optimal information size. |
| CD4+ T-cell count | Publication bias | Not assessed | Not assessed by funnel-plot methods because only 7 trial(s) contribute, below the usual minimum of 10. Small-study effects cannot be excluded; no additional downgrade was applied. |

Certainty was assessed at construct level using the primary final-value evidence for inflammatory biomarkers and the full unstandardized mean-difference evidence for CD4+ T-cell count. The primary inflammatory bodies consist exclusively of randomized trials and start at high certainty. CD4+ T-cell count includes six randomized trials and one non-randomized trial carrying 19.7% of the random-effects weight; it also starts at high certainty on the randomized evidence, while the serious ROBINS-I judgment is reflected in risk of bias. No construct was downgraded for indirectness: all comparators met the prespecified target contrast, and any placebo, medication, or care background was applied equally to both arms. The recreationally active comparator was examined in a post hoc robustness analysis.

Publication bias was not assessed for any construct because fewer than 10 trials contributed, below the recommended minimum for funnel-plot or regression-based assessment. Small-study effects therefore cannot be excluded, but no additional downgrade was applied solely because formal assessment was impossible.

**Table S7. Constructs reported but not pooled, and the reason for narrative synthesis.**

| **Outcome** | **Domain** | **Trials with a poolable value** | **Trials reporting the construct** | **Reason for not pooling** |
| --- | --- | --- | --- | --- |
| Adiponectin | Other prespecified outcomes | 2 | Ghayomzadeh et al. (2022); Yarasheski et al. (2011) | Prespecified for narrative synthesis. 2 trial(s) provided a poolable numeric contrast, but this secondary outcome was not included in the quantitative primary-outcome synthesis. |
| Brain-derived neurotrophic factor (BDNF) | Other prespecified outcomes | 0 | Khoramipour et al. (2022) | No trial provides a poolable numeric contrast; the only source values are figure-based and could not be recovered to poolable precision. |
| CD4+ T-cell percentage | Other prespecified outcomes | 1 | Gomes et al. (2010) | Prespecified for narrative synthesis. 1 trial(s) provided a poolable numeric contrast, but this secondary outcome was not included in the quantitative primary-outcome synthesis. |
| CD8+ T-cell count | Other prespecified outcomes | 2 | Soares et al. (2022); Zanetti et al. (2016) | Prespecified for narrative synthesis. 2 trial(s) provided a poolable numeric contrast, but this secondary outcome was not included in the quantitative primary-outcome synthesis. |
| Cortisol | Other prespecified outcomes | 2 | Dudgeon et al. (2012); Vingren et al. (2018) | Prespecified for narrative synthesis. 2 trial(s) provided a poolable numeric contrast, but this secondary outcome was not included in the quantitative primary-outcome synthesis. |
| D-dimer | Other prespecified outcomes | 1 | Piraux et al. (2019) | Prespecified for narrative synthesis. 1 trial(s) provided a poolable numeric contrast, but this secondary outcome was not included in the quantitative primary-outcome synthesis. |
| Fibrinogen | Other prespecified outcomes | 1 | Zanetti et al. (2020) | Prespecified for narrative synthesis. 1 trial(s) provided a poolable numeric contrast, but this secondary outcome was not included in the quantitative primary-outcome synthesis. |
| Granulocyte-macrophage colony-stimulating factor (GM-CSF) | Other prespecified outcomes | 1 | Pedro et al. (2017) | Prespecified for narrative synthesis. 1 trial(s) provided a poolable numeric contrast, but this secondary outcome was not included in the quantitative primary-outcome synthesis. |
| Growth hormone (GH) | Other prespecified outcomes | 1 | Dudgeon et al. (2012) | Prespecified for narrative synthesis. 1 trial(s) provided a poolable numeric contrast, but this secondary outcome was not included in the quantitative primary-outcome synthesis. |
| HIV-1 viral load | Other prespecified outcomes | 0 | Khoramipour et al. (2022); Piraux et al. (2019); Soares et al. (2022); Zanetti et al. (2016) | Prespecified as narrative: viral load is reported as undetectable or left-censored below the assay limit in every contributing trial, so no continuous contrast exists. |
| Insulin-like growth factor 1 (IGF-1) | Other prespecified outcomes | 2 | Dudgeon et al. (2012); Ghayomzadeh et al. (2022) | Prespecified for narrative synthesis. 2 trial(s) provided a poolable numeric contrast, but this secondary outcome was not included in the quantitative primary-outcome synthesis. |
| Insulin-like growth factor-binding protein 3 (IGFBP-3) | Other prespecified outcomes | 1 | Dudgeon et al. (2012) | Prespecified for narrative synthesis. 1 trial(s) provided a poolable numeric contrast, but this secondary outcome was not included in the quantitative primary-outcome synthesis. |
| Interferon gamma (IFN-γ) | Other prespecified outcomes | 2 | Pedro et al. (2017); Vingren et al. (2018) | Prespecified for narrative synthesis. 2 trial(s) provided a poolable numeric contrast, but this secondary outcome was not included in the quantitative primary-outcome synthesis. |
| Interleukin-18 (IL-18) | Other prespecified outcomes | 1 | Ryan et al. (2025) | Prespecified for narrative synthesis. 1 trial(s) provided a poolable numeric contrast, but this secondary outcome was not included in the quantitative primary-outcome synthesis. |
| Interleukin-2 (IL-2) | Other prespecified outcomes | 1 | Vingren et al. (2018) | Prespecified for narrative synthesis. 1 trial(s) provided a poolable numeric contrast, but this secondary outcome was not included in the quantitative primary-outcome synthesis. |
| Interleukin-4 (IL-4) | Other prespecified outcomes | 2 | Pedro et al. (2017); Vingren et al. (2018) | Prespecified for narrative synthesis. 2 trial(s) provided a poolable numeric contrast, but this secondary outcome was not included in the quantitative primary-outcome synthesis. |
| Interleukin-5 (IL-5) | Other prespecified outcomes | 1 | Pedro et al. (2017) | Prespecified for narrative synthesis. 1 trial(s) provided a poolable numeric contrast, but this secondary outcome was not included in the quantitative primary-outcome synthesis. |
| Irisin | Other prespecified outcomes | 1 | Ghayomzadeh et al. (2022) | Prespecified for narrative synthesis. 1 trial(s) provided a poolable numeric contrast, but this secondary outcome was not included in the quantitative primary-outcome synthesis. |
| Leptin | Other prespecified outcomes | 1 | Pedro et al. (2017) | Prespecified for narrative synthesis. 1 trial(s) provided a poolable numeric contrast, but this secondary outcome was not included in the quantitative primary-outcome synthesis. |
| Myostatin | Other prespecified outcomes | 1 | Ghayomzadeh et al. (2022) | Prespecified for narrative synthesis. 1 trial(s) provided a poolable numeric contrast, but this secondary outcome was not included in the quantitative primary-outcome synthesis. |
| Soluble tumor necrosis factor receptor 2 (sTNFR2) | Other prespecified outcomes | 1 | Dudgeon et al. (2012) | Prespecified for narrative synthesis. 1 trial(s) provided a poolable numeric contrast, but this secondary outcome was not included in the quantitative primary-outcome synthesis. |
| Testosterone | Other prespecified outcomes | 1 | Dudgeon et al. (2012) | Prespecified for narrative synthesis. 1 trial(s) provided a poolable numeric contrast, but this secondary outcome was not included in the quantitative primary-outcome synthesis. |
| Vascular cell adhesion molecule 1 (VCAM-1) | Other prespecified outcomes | 1 | Vingren et al. (2018) | Prespecified for narrative synthesis. 1 trial(s) provided a poolable numeric contrast, but this secondary outcome was not included in the quantitative primary-outcome synthesis. |

The outcomes in Table S7 were prespecified secondary outcomes for narrative synthesis, regardless of how many trials reported them. The column 'Trials with a poolable value' records whether a numeric contrast and variance could be recovered; it does not imply that a meta-analysis was planned for these secondary outcomes.

HIV-1 viral load was prespecified for narrative synthesis: every contributing trial reports it as undetectable or below the assay limit, so no continuous contrast exists.

Khoramipour et al. (2022) reports its outcomes only as bar charts without numerical values or dispersion, so no estimate could be recovered to poolable precision and the trial is described narratively throughout. The C-reactive protein values of Zanetti et al. (2016) are reported in the same way and are likewise not pooled.

BDNF, brain-derived neurotrophic factor; CRP, C-reactive protein; GM-CSF, granulocyte-macrophage colony-stimulating factor; HIV, human immunodeficiency virus; IFN-γ, interferon gamma; IGF-1, insulin-like growth factor 1; IGFBP-3, insulin-like growth factor-binding protein 3; IL, interleukin; sTNFR2, soluble tumor necrosis factor receptor 2; VCAM-1, vascular cell adhesion molecule 1.

**Supplementary Figures**

**Figure S1. Primary final-value forest plot of Interleukin-6 (IL-6).**

Final-value standardized mean differences (Hedges' g) are the primary evidence and are shown as exercise minus comparator with 95% confidence intervals. Squares are trial estimates with area proportional to random-effects weight. When at least two trials contribute, the diamond is the pooled random-effects estimate; a dashed line is the 95% prediction interval when at least three trials contribute. Pooling uses restricted maximum likelihood and a Hartung-Knapp-Sidik-Jonkman confidence interval. n exp/ctrl, number analyzed in the exercise and comparator groups; Wt, weight; CI, confidence interval; I², heterogeneity statistic; SMD, standardized mean difference; τ², between-study variance.

**Figure S2. Primary final-value forest plot of Tumor necrosis factor alpha (TNF-α).**

Final-value standardized mean differences (Hedges' g) are the primary evidence and are shown as exercise minus comparator with 95% confidence intervals. Squares are trial estimates with area proportional to random-effects weight. When at least two trials contribute, the diamond is the pooled random-effects estimate; a dashed line is the 95% prediction interval when at least three trials contribute. Pooling uses restricted maximum likelihood and a Hartung-Knapp-Sidik-Jonkman confidence interval. n exp/ctrl, number analyzed in the exercise and comparator groups; Wt, weight; CI, confidence interval; I², heterogeneity statistic; SMD, standardized mean difference; τ², between-study variance.

**Figure S3. Primary final-value forest plot of C-reactive protein (CRP).**

Final-value standardized mean differences (Hedges' g) are the primary evidence and are shown as exercise minus comparator with 95% confidence intervals. Squares are trial estimates with area proportional to random-effects weight. When at least two trials contribute, the diamond is the pooled random-effects estimate; a dashed line is the 95% prediction interval when at least three trials contribute. Pooling uses restricted maximum likelihood and a Hartung-Knapp-Sidik-Jonkman confidence interval. n exp/ctrl, number analyzed in the exercise and comparator groups; Wt, weight; CI, confidence interval; I², heterogeneity statistic; SMD, standardized mean difference; τ², between-study variance.

**Figure S4. Primary final-value forest plot of Interleukin-1 beta (IL-1β).**

Final-value standardized mean differences (Hedges' g) are the primary evidence and are shown as exercise minus comparator with 95% confidence intervals. Squares are trial estimates with area proportional to random-effects weight. When at least two trials contribute, the diamond is the pooled random-effects estimate; a dashed line is the 95% prediction interval when at least three trials contribute. Pooling uses restricted maximum likelihood and a Hartung-Knapp-Sidik-Jonkman confidence interval. n exp/ctrl, number analyzed in the exercise and comparator groups; Wt, weight; CI, confidence interval; I², heterogeneity statistic; SMD, standardized mean difference; τ², between-study variance.

**Figure S5. Individual final-value result for Interleukin-8 (IL-8).**

The Primary final-value evidence for interleukin-8 consists of one trial and retains its Primary role despite not being pooled. The square is the individual study standardized mean difference (Hedges' g) with its 95% confidence interval. No pooled diamond, heterogeneity statistic, or prediction interval is shown because a one-trial stratum is not a meta-analysis. n exp/ctrl, number analyzed in the exercise and comparator groups; CI, confidence interval; SMD, standardized mean difference.

**Figure S6. Primary final-value forest plot of Interleukin-10 (IL-10).**

Final-value standardized mean differences (Hedges' g) are the primary evidence and are shown as exercise minus comparator with 95% confidence intervals. Squares are trial estimates with area proportional to random-effects weight. When at least two trials contribute, the diamond is the pooled random-effects estimate; a dashed line is the 95% prediction interval when at least three trials contribute. Pooling uses restricted maximum likelihood and a Hartung-Knapp-Sidik-Jonkman confidence interval. n exp/ctrl, number analyzed in the exercise and comparator groups; Wt, weight; CI, confidence interval; I², heterogeneity statistic; SMD, standardized mean difference; τ², between-study variance.

**Figure S7. Subgroup analyses for CD4+ T-cell count.**

The diamond is the estimate across all contributing CD4+ trials and squares are estimates within subgroup levels, each with a 95% confidence interval. The p value beside each moderator is the Cochran test for a difference between levels. These subgroup analyses were post hoc exploratory and were restricted to moderators with at least three trials in every level. k, number of trials; CI, confidence interval.

**Figure S8. Post hoc robustness analyses for the revised primary and supporting estimates.**

Circles are meta-analytic estimates with 95% confidence intervals; squares denote one-trial individual results that were not pooled. The dashed vertical line marks the Primary estimate only, not a common reference for differently standardized forms; the gray line marks no effect. All robustness analyses are post hoc exploratory. Inflammatory final-value and change-score rows are Primary and Supporting references, respectively; other rows are Sensitivity checks of the corresponding reference analysis, not comparisons between forms. The CD4+ final-value-only and change-score-only rows are Sensitivity analyses of its combined-form Primary estimate. k, number of trials; CI, confidence interval; MD, mean difference; SMD, standardized mean difference.

**Figure S9. Availability of outcome data across trials and constructs.**

Filled squares indicate a Primary or Supporting quantitative estimate, including unpooled individual results; open circles indicate a usable value in a Narrative construct; crosses indicate a reported outcome without a recoverable numeric contrast. Filled squares do not distinguish Primary from Supporting roles: inflammatory final values are Primary and change scores Supporting, whereas both CD4+ forms enter its Primary analysis. The horizontal rule separates the seven quantitatively assessed constructs (above) from the 23 Narrative constructs (below).
